# Integrating Genetic Mapping and BSR-Seq Analysis to Identify Candidate Genes Controlling Fruitfulness in *Camellia sinensis*

**DOI:** 10.3390/plants14192963

**Published:** 2025-09-24

**Authors:** Shizhuo Kan, Dandan Tang, Wei Chen, Yuxin Gu, Shenxin Zhao, Lu Long, Jing Zhang, Xiaoqin Tan, Liqiang Tan, Qian Tang

**Affiliations:** 1 College of Horticulture, Sichuan Agricultural University, Chengdu 611130, China; kanruoyuan@163.com (S.K.); tddtea11@163.com (D.T.); chenwei2551@163.com (W.C.); 19936682334@163.com (Y.G.); shenxinshenxinshen@126.com (S.Z.); ll18385862840@126.com (L.L.); zhzhjj12345@163.com (J.Z.); xqintan@163.com (X.T.); 2 Tea Resources Utilization and Quality Testing Key Laboratory of Sichuan Province, Sichuan Agricultural University, Chengdu 611130, China

**Keywords:** tea plant, fruit number, QTL mapping, BSR-seq, *CsETR2*

## Abstract

As nutrient allocation trade-offs occur between reproductive and vegetative development in crops, optimizing their partitioning holds promise for improving agricultural productivity and quality. Herein, we characterize the phenotypic diversity of the fruitfulness trait and identify associated genes in tea plants (*Camellia sinensis*). Over three consecutive years, we monitored the fruitfulness of an F_1_ hybrid population (*n* = 206) derived from crosses of ‘Emei Wenchun’ and ‘Chuanmu 217’. A marked variation was observed in the yield of individual plants, ranging from complete sterility (zero fruits) to exceptionally high fertility (1612 fruits). Using the high-resolution genetic linkage map and the fruitfulness data, we identified a stable major QTL designated as *qFN5*. To fine-map the underlying gene(s), artificial pollination experiments were conducted with extreme phenotype individuals (with the highest vs. lowest fruit numbers). Bulked segregant RNA sequencing (BSR-Seq) with ovules collected at two and seven days post-pollination (DPP) identified the genomic intervals that exhibit a high degree of overlap with *qFN5*. Analysis of expression dynamics combined with functional genomics data revealed a prominent candidate gene, *CsETR2* (TGY048509), which encodes an ethylene receptor protein. When *CsETR2* was overexpressed in *Arabidopsis thaliana*, the transgenic lines exhibited significantly decreased reproductive performance relative to the wild-type plants. Relative to the wild type, the transgenic lines exhibited a significant decline in several key traits: the number of effective panicles decreased by 72.5%, the seed setting rate dropped by 67.7%, and the silique length shortened by 38%. These findings demonstrate its role in regulating plant fruitfulness. Furthermore, yeast one-hybrid and dual-luciferase assays verified that *CsMYB15* (TGY110225) directly binds to the *CsETR2* promoter, thus repressing its transcription. In summary, our findings expand the understanding of genetic regulation underlying fruitfulness in tea plants and provide candidate target loci for breeding.

## 1. Introduction

Tea plant (*Camellia sinensis* (L.) O. Kuntze) is a perennial evergreen plant widely cultivated around the world. The continuous genetic improvement of tea plants is an important task for major tea-producing countries such as China and India [1], which poses a challenge to tea plant germplasm innovation and genetic breeding. In order to accelerate the tea plant breeding process and meet the ever-changing demands of the tea industry, predecessors have screened and identified some resources with unique biochemical components and phenotypic traits through breeding methods such as hybridization [2,3]. However, the breeding methods for tea plants, the genetic mapping of key agronomic traits, and gene mining are still progressing slowly.

Flowering and fruit number are two of the most important agronomic traits of the tea plant. For cultivated tea plants, fewer or even no flowers and fruit numbers are more advantageous, as this reduces the consumption of nutrients by reproductive growth; however, for hybrid breeding and genetic studies, a greater number of flowers and fruit numbers, especially a higher fruit number rate, is more beneficial. In addition to the tender new shoots being harvested for tea production, the flowers and fruit of the tea plant have also been found to possess significant application value and are increasingly valued by researchers and cultivators. Therefore, the study of the fruit number traits of the tea plant has multiple implications.

Under the same environmental conditions, there is a significant difference in the fruit number ability among different tea plant varieties. This variation is characterized by a range of hundreds of times in the number of fruits produced by individual plants. Genetics is the key factor influencing the number of fruits. However, the tea plant has a long fruiting cycle (it takes about 450 days from flower to bud differentiation to seed maturity), making it difficult to discover genes affecting fruiting through reverse genetics. Only a few studies have approached this issue by Quantitative Trait Locus (QTLs). To verify the application of high-density genetic maps, Wei et al. utilized the F_1_ population from the cross between ‘Longjing 43’ and ‘Baihaozao’ to map the fruit number trait, identifying four overlapping QTLs that were mapped to chromosomes LG1, 2, 5, and 15, respectively [2]. Wang et al. identified 10 QTLs by statistically analyzing the fruit setting phenotype data of the ‘Longjing 43’ × ‘Baihaozao’ hybrid F_1_ population, combined with QTL mapping analysis and genome comparison, and preliminarily identified 11 key candidate genes [4]. However, the genetic control of this critical trait is largely unclear, with very little related research conducted, and the genetic basis and mode of inheritance of this trait are largely unknown.

In a multi-year study spanning 2021–2023, we conducted a comprehensive evaluation of the F_1_ hybrid population from the ‘Emei Wenchun’ (EW) × ‘Chuanmu 217’ (CM) cross. Our analysis demonstrated a substantial and statistically significant difference in fruit set rate among the individuals of this population. The number of fruits per plant ranges from a minimum of 0 to a maximum of 1612. Therefore, we utilized three years of observational data in conjunction with the high-density genetic map constructed by our research group and the high-quality reference genomes of some tea plant varieties that have been published [5,6,7]. In this study, we integrated two complementary approaches, QTL mapping and BSR-Seq, to systematically identify the genetic loci controlling fruiting traits. This strategy aimed to uncover the primary molecular factors underlying these phenotypic variations. This research aims to provide a reference for the selection of high-quality individuals and further directional breeding, and it can also offer theoretical support for balancing vegetative and reproductive growth.

## 2. Materials and Methods

### 2.1. Experimental Materials

The mapping population used in this study consists of the natural hybrid F_1_ generation (*n* = 206) from the maternal parent EW and the paternal parent CM. In our previous study, reduced-representation genome sequencing and paternal identification were conducted on this progeny population, identifying 217 hybrid progeny derived from a cross between EW and CM. Utilizing this full-sib population, a high-density genetic map of the tea plant was constructed, containing 4244 single nucleotide polymorphism (SNP) markers with an average genetic distance of 0.34 cM [1]. This group is cultivated in Mingshan County, Sichuan Province, China (N 28°59′, E 103°53′). This study primarily focuses on the 206 hybrid progeny that demonstrate optimal growth and convenient measurement for field observation and artificial pollination. During the trial period, the field management of tea plants, including fertilization and pruning, was maintained at a consistent level. The fertilization and pruning during the trial period adopted conventional cultivation management measures for productive tea gardens, including one basal dressing (in November), two top dressings (in February and July), and two light prunings (in June and November). All plants within the population were subjected to uniform cultural practices throughout the experimental period.

### 2.2. Experimental Method

#### 2.2.1. Phenotypic Data Collection

During the period from 2021 to June 2023, a statistical analysis was conducted on the fruit number (FN) of the F_1_ population consisting of 206 hybrid progeny of the EW × CM. To ensure counting accuracy, all fruits from each tea plant were harvested, collected in uniquely labeled bags, and subsequently quantified.

#### 2.2.2. Data Statistics and Analysis

Statistical analysis was performed with Microsoft Excel 2016. Phenotypic data, including fruit number from a full-sib progeny, were analyzed using descriptive statistics (mean, range, SD, and CV) to quantify variation. The distribution of fruit number was visualized with a histogram.

#### 2.2.3. QTL Mapping Analysis

The QTL mapping for fruit number traits was performed using MapQTL6.0 software [8]. Combining the genetic map constructed by the research group in the early stage using SNP genetic markers with the fruit number data of the offspring populations from 2021, 2022, and 2023, Interval Mapping (IM) was employed for QTL mapping analysis. Using LOD = 4 as the threshold, loci with LOD ≥ 4 are considered as a QTL. The QTL mapping figures are drawn using the Mapchart 2.32 software [9]. QTL naming principles refer to the principles of the research group: q-trait English abbreviation-chromosome number observation year. “*qFN5*_2021” indicates that the QTL mapped to chromosome 5 (chr5) of the tea plant fruiting total in the 2021 mapping population.

#### 2.2.4. Hybrid Pollination Treatment and Sampling

Based on the fruit number data of the progeny tea plants from 2021 to 2023 counted in Section 2.2.2, 10 individual plants with extremely high (FNH) and extremely low (FNL) average fruit number rates were selected as materials for BSR-seq. Artificial pollination was performed on the parents and progeny in the mixed pool in late October 2023. Harvesting the buds of tea plants during the bud-white stage, placing the flowers into the oven, setting the oven to about twenty-five degrees, and conducting multiple flips during the drying process. Using a brush to gently brush the pollen from the stamens. Pollination is carried out on clear days, and bags are quickly applied after pollination. Subsequently, continuously collected the ovary samples from the parent plants and each individual plant in the mixed pool on the second and seventh day after pollination. The samples are then flash-frozen in liquid nitrogen and stored in a −80 °C freezer for the construction of mixed pools and transcriptome sequencing.

#### 2.2.5. Pollen Tube Fluorescence Microscopy Observation

The pollinated flowers were collected 6 h, 24 h, 48 h, and 72 h after pollination, fixed in FAA solution, and stored at 4 °C. At least 20 pistils for microscopic examination were prepared [10]. The outer floral layers were carefully removed to isolate the pistils. (1) Water seepage: The fixed and preserved pistils were subjected to sequential immersion in 70% ethanol for 40 min, 50% ethanol for 30 min, and 30% ethanol for 30 min. Following this, the pistils were rinsed three times in distilled water, with each rinse lasting 3 min. (2) Softening: The pistils were subsequently softened in an 8 mol/L NaOH solution at 40 °C for 8 h. (3) After removal, the pistils were rinsed three times with distilled water, each rinse lasting 3 min. (4) Staining: Pistils were softened and stained with 0.1% aniline blue in 0.1 NK_3_PO_4_ for 12 h in the dark. (5) Pressing and observation: The stained pistils were mounted on a slide, a drop of 50% glycerol was applied, and a coverslip was placed over the pistils. Gentle pressing with forceps was performed to disperse the pistils evenly. Pollen germination on the stigma surface and pollen tube growth within the pistils were observed under a fluorescence microscope (country of origin: Germany; manufacturer: Zeiss; DAPI blue: excitation wavelength: 365 nm; emission wavelength range: 420–470 nm), and micrographs were captured accordingly [11].

#### 2.2.6. BSR-Seq Analysis

##### Sequence Data Analysis

We obtained eight transcriptome samples, consisting of four parental samples and four pooled samples, with three biological replicates for each. All experimental procedures, including RNA extraction, quality assessment, pooled sequencing, and differential gene expression analysis, were performed by Metawell Biotechnology Co., Ltd. (Wuhan, China). After the library was constructed, quantitative PCR (q-PCR) was used to accurately quantify the effective concentration of the library (effective concentration > 2 nM). After the library passed the quality control checks and met the required standards, sequencing was conducted on the Illumina platform. We conducted stringent quality control on the data using fastp [1]. The filtering criteria were as follows: adapter-containing reads were removed; paired reads were discarded if the proportion of N bases in either read exceeded 10%; and paired reads were discarded if the proportion of low-quality bases (Q-value ≤ 20) in either read exceeded 50%. Transcriptome sequencing of 24 samples yielded 282.14 Gb of clean data, with each sample having ≥ 6 Gb and a Q30 base ratio ≥ 93%. Clean data were aligned to the ‘Tieguanyin’ tea plant genome using HTISAT2 (v2.0.4). Initial assembly of genes/transcripts was performed via StringTie (Stringtie-1.3.4d. Linux_x86_64), and results from all samples were merged. Gffcompare (gficompare-0.9.8. Linux_x86_64) was used to compare transcripts with reference annotations for final assembly annotation. FPKM quantification was performed using the ballgown package with the following formula: FPKM = (total exon fragments/mapped reads (millions)) × exon length (kb).

##### Differential Expression Analysis and Enrichment Analysis

The differential expression analysis between sample groups was performed using the R package DESeq2 (R4.1.2) [6,12]. Genes with|log2Fold Change| ≥ 1 and FDR (False Discovery Rate) < 0.05 were defined as differentially expressed genes (DEGs, differentially expressed genes). These genes were annotated using the Kyoto Encyclopedia of Genes and Genomes (KEGG) (http://www.kegg.jp/ (accessed on 23 December 2023)).

##### BSR-Seq Analysis for Fruit Number Traits

Two algorithms were used to locate the association intervals for fruit number traits, namely the G′ value and OcBSA methods. The R package QTLseqr was used for the G′ value method localization [7]. Prior to analysis, SNP sites in the mixed pool were filtered based on parental data (sites that are homozygous in both parents were removed), and after data input, further filtering was performed to retain sites with allele frequencies between 0.1 and 0.9 and sequencing depths between 10 and 300. Subsequently, the runGprimeAnalysis function is used to perform the G′ value analysis, with a sliding window size of 1 × 10^7^, while keeping the other parameters unchanged. When using the OcBSA software for localization, the sequencing depth for the mixed pool is set to 10~300, the sliding window size is 1 × 10^7^, and the other parameters remain default [13].

#### 2.2.7. Combined Analysis of QTL and BSR-Seq

Based on QTL mapping and BSR sequencing data, we identified overlapping chromosome regions. We compared gene sequences in this interval with those in the Tea plant Genome Database (TeaTGDB) (http://eplant.njau.edu.cn/tea/index.html) (accessed on 23 December 2023), confirmed all genes within this region, and checked their functional annotations on the Kyoto Encyclopedia of Genes and Genomes (KEGG) database. Finally, based on related literature reports, we filtered out potential candidate genes.

#### 2.2.8. Validation of Differential Genes by qRT-PCR

For the candidate genes, the relative expression levels were validated using qRT-PCR technology. Design four specific primers for four differential genes using Primer-BLAST (Table S1). In the qRT-PCR experiment, we adhered strictly to standard protocols, and each sample included 3 biological replicates and 3 technical replicates. Total RNA was extracted from the tea ovary using the OMEGA Plant RNA Kit (Omega Bio-Tek, Norcross, GA, USA) following the kit protocol. The RNA concentration and purity were measured using a Nanodrop One spectrophotometer (Thermo Scientific, Waltham, MA, USA), and RNA integrity was assessed via 1% agarose gel electrophoresis. RNA reverse transcription was performed using the Taraka PrimeScript™ FAST RT Kit with gDNA Eraser (Bao Biological Engineering, Dalian, China), with a maximum RNA input of 1 μg per 10 μL reaction volume. qRT-PCR were performed using “Takara TB Green Premix Ex Taq II FAST qPCR”. 10 μL reaction system was employed, consisting of 5 μL of 2 × SYBR Green PCR Master Mix, 0.4 μL of each forward and reverse primer (10 μM), 2 μL of cDNA, and 2.2 μL of ddH_2_O. *GAPDH* was selected as the reference gene, and the relative expression levels of the genes were calculated using the 2^−ΔΔCT^ method [4,14]. GraphPad Prism 10.1.2 and Tbtools 2.154 software were used for data visualization.

#### 2.2.9. Functional Analysis of the *CsETR2*

##### Overexpression of *CsETR2* in *Arabidopsis thaliana*

Overexpression analyses were conducted for four candidate genes, among which the overexpression of *CsETR2* in *Arabidopsis thaliana* exhibited relatively distinct phenotypes. Therefore, subsequent experiments were carried out on this gene. The CDS of *CsETR2*, obtained through PCR amplification, is 2292 bp long and encodes 763 amino acids. Similarly, the reference genome sequence TGY048509 has an identical CDS length of 2292 bp and encodes the same number of amino acids. *CsETR2* was cloned into the pCAMBIA1300-35S-EGFP expression vector, and the correctly sequenced overexpression vector was transformed into Agrobacterium (Table S1), Subsequently introduced into *Arabidopsis thaliana* (WT, Col-1) using the floral dip method [15] until T_2_ generation seeds were harvested. *CsETR2* transgenic plants were screened on a medium containing 30 mg/L hygromycin (1/2MS). When the T_2_ generation transgenic seedlings grew to 5 weeks, detection primers were designed to confirm that the overexpression vector had been successfully introduced into *Arabidopsis thaliana*. Meanwhile, after validation through qRT-PCR analysis, T_2_-positive lines were obtained for further analysis. WT seeds and transgenic seeds were sterilized with 75% ethanol for 5 min, washed with ddH_2_O 5–8 times, then sterilized with 5–10% sodium hypochlorite for 5 min, washed with ddH_2_O 5–8 times, vernalized at 4 °C for 2–3 days, and sown on 1/2 MS medium and 1/2 MS medium containing 30 mg/L hygromycin, respectively. Then, transfer the culture medium plants to a 21 °C incubator for germination under a 16 h/8 h light/dark cycle. After 7–10 days of germination, transplant the plants into soil, return them to the incubator, and manage with regular watering.

##### Yeast One-Hybrid Assay

The transcription factor binding site sequences were tandemly repeated, and the pAbAi vector was constructed using the homologous recombination method (Table S1). The constructed pAbAi vector was subjected to single enzyme digestion with BstBI, and the linearized vector was then transformed into Y1HGold yeast cells, which were spread on SD/-Ura solid medium and cultured at 28 °C for 2–3 days. Colonies with standard growth morphology and uniform size were picked for positive colony PCR identification. The positive colonies were resuspended to achieve standardized cell densities (OD600 = 0.2, 0.02, 0.002, 0.0002), followed by verification of transcriptional self-activation of the bait strain (the concentrations of Aureobasidin A (AbA) were 0, 300, 500, 700, and 900 ng/mL), cultured at 28 °C for 2 to 3 days. The transcription factor was constructed into the pGADT7 vector, and positive colonies were verified by sequencing. The constructed pAbAi vector and pGADT7 vector were used for the preparation and transformation of yeast competent cells, with the empty pGADT7 vector serving as the negative control. The interaction was determined by systematically evaluating the development of colonies after cultivation.

##### Dual-Luciferase Assay

The coding sequence of the transcription factor was cloned into the pGreenII 62-SK vector driven by the 35S promoter, while the reporter gene was inserted into the pGreenII 0800-Luc vector. The vectors confirmed by sequencing were used for transformation with *Agrobacterium tumefaciens* GV3101 (pSoup-p19). The identified positive Agrobacterium strains were injected into *Nicotiana tabacum* leaves, with the experimental group and control group being injected into different regions of the same leaf, respectively. Two days later, the abaxial side of the leaves was sprayed with a 1 mM D-luciferin sodium salt solution then placed into an in vivo imaging system to collect and analyze the luciferase signals. Quantitative analysis was performed using the Double Luciferase Reporter Assay Kit (Wuhan, China) from Wuhan Genomix Biotech Co., Ltd. The firefly luciferase (LUC) activity values were normalized using the Renilla luciferase (REN) internal reference signal (expressed as the LUC/REN ratio) and standardized against the control group.

## 3. Results

### 3.1. Tea Plant Fruit Number Related Parameter Phenotypic Data Collection

**Figure 1:**
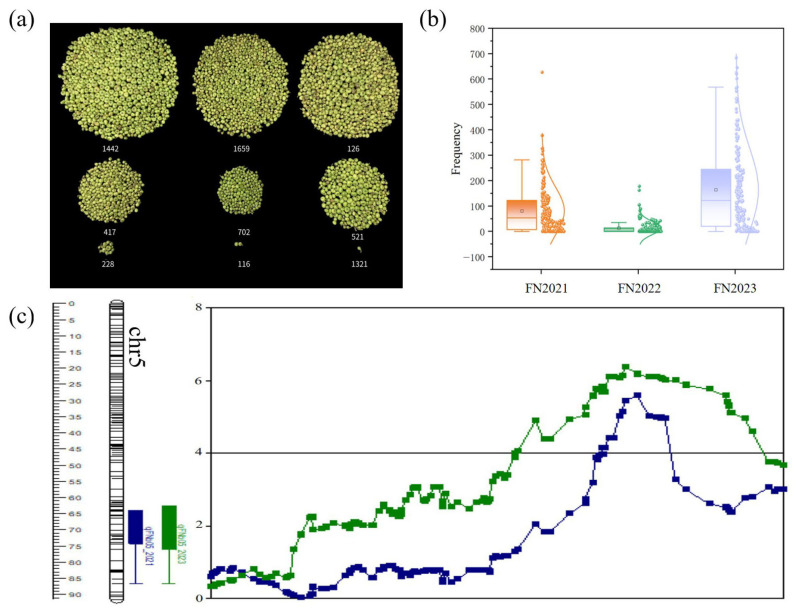
Comparison of differences in the number of fruits per plant in the F_1_ generation (partial), frequency distribution of tea fruit number, and QTL mapping. (**a**) Comparison of differences in FN (partial). (**b**) 2021–2023 Frequency Distribution Chart of FN. (**c**) QTL mapping, the blue area represents QTLs related to fruit number traits in 2021, and the green area represents QTLs related to fruit number traits in 2023.

Statistical analysis was conducted on the fruit quantity of 206 F_1_ populations. The results indicated (Table S2) that the number of fruit per plant in this population ranges from 0 to 627, 0 to 178, and 0 to 1612 annually. Phenotypic analysis of fruit number per plant across three consecutive years (2021–2023) revealed considerable variation (Table S3). In 2021, the fruit number ranged from 0 to 627, with an average of 80.48 and a coefficient of variation of 116.69%; for the year 2022, the fruit number per plant ranged from 0 to 178, with an average of 13.61 and a coefficient of variation of 187.22%. Finally, in 2023, the fruit number per plant ranged from 0 to 1612, with an average of 171.19 and a coefficient of variation of 112.37%. The survey data over three years indicate that there is a significant variation in the number of fruit among the tea plant population, with coefficients of variation exceeding 100%, suggesting a strong potential for variability in tea plant fruit number. Representative progeny were selected to illustrate the range of phenotypic variation in fruit number. As depicted in Figure 1a, there is a significant difference in the number of fruits. Progeny with identification numbers 126, 1442, and 1659 have fruit numbers that are a hundredfold more than those with numbers 116, 228, and 1321 (Figure 1a). Furthermore, from the frequency distribution histogram of tea fruit over the years (Figure 1b), it can be observed that the data related to tea fruit number in this group all exhibit a skewed distribution trend, which aligns with the genetic characteristics of quantitative traits. The obtained data can be used for QTL mapping.

### 3.2. Analysis of QTL Mapping Results

Among the three years of continuously collected phenotypic data for fruit number, the same QTL was located in both 2021 and 2023, positioned on chromosome 5 (Figure 1c and Table S4), and named *qFN5*_2021 and *qFN5*_2023, respectively. The *qFN5*_2021 locus LOD scores are 5.83 for markers Marker217646-Marker221210, with a genetic distance of 8.60 cM and a physical interval of 158216749–178312926 (20.10 cM), explaining 11.8% of the phenotypic variation (PVE). The LOD score for the *qFN5*_2023 locus is 6.38, with the flanking marker extension interval being Marker217263-Marker221745, the genetic distance is 13.47 cM, and the physical interval is 143810774–182412304 (38.6 cM), which can explain 13.3% of the phenotypic variation (PVE) (Table S5). The positioning effects of *qFN5*_2021 and *qFN5*_2023 over the two years have been relatively good, and the interval ranges overlap (Figure 1c). The QTL positioning results for the fruit number data in 2022 were not satisfactory, with no QTL meeting the LOD threshold conditions and PVE ≥ 10%.

### 3.3. BSR-Seq Result Analysis

#### 3.3.1. Fluorescence Microscopic Observation of Pollen Tube Germination and BSR-Seq Sequencing Data Analysis

**Figure 2:**
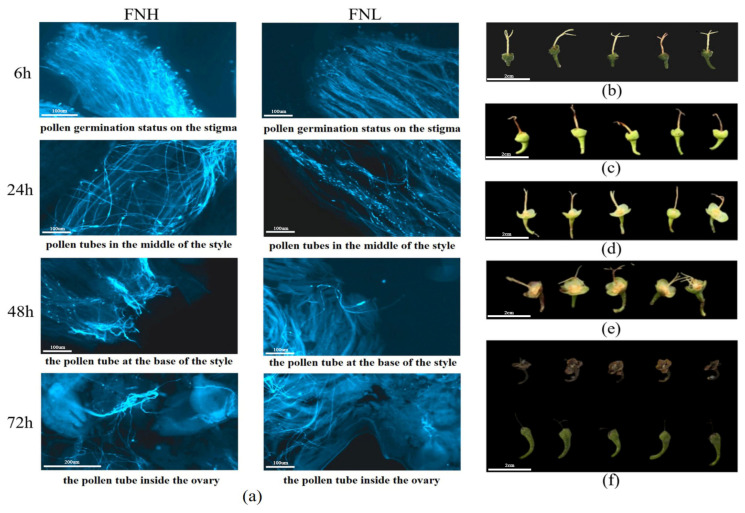
Pollen tube germination was observed under a fluorescence microscope, and developmental status was observed after pistil pollination. (**a**) Fluorescence microscopy image of pollen tube germination; (**b**) and (**c**) represent the growth status of FNH at 2 days and 7 days post-pollination, respectively; (**d**) and (**e**) represent the growth status of FNL at 2 days and 7 days post-pollination, respectively; (**f**) The following year in March, the pistils of normal development and non-development.

**Figure 3:**
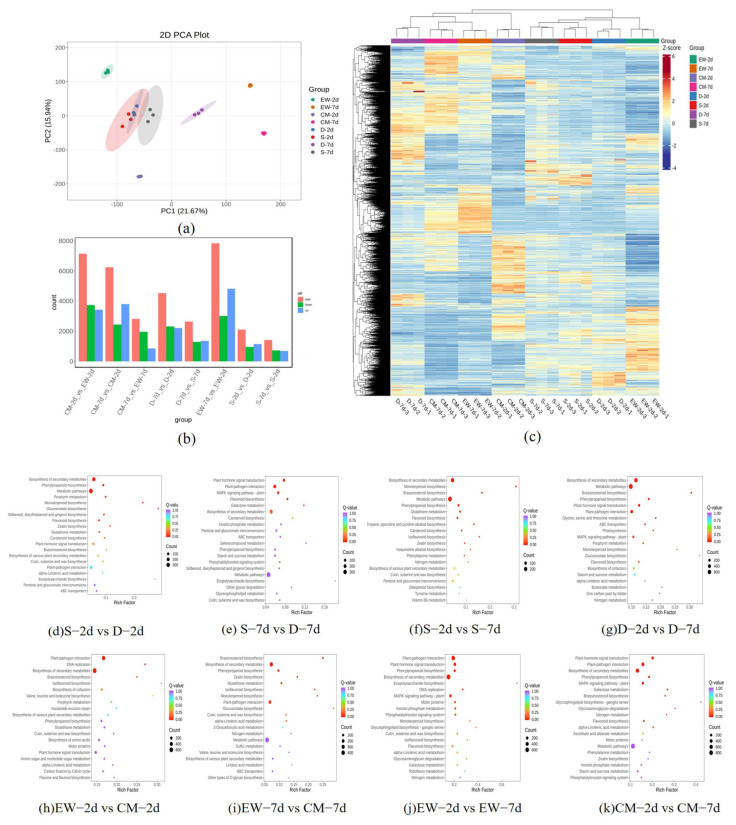
Correlation graph of parental and extreme offspring single plant samples and KEGG enrichment of differentially expressed genes in each comparison group. (**a**) PCA clustering diagram; (**b**) differential expression gene bar chart; (**c**) differential expression gene clustering heatmap; (**d**–**k**) KEGG enrichment.

Using a fluorescence microscope to observe the growth of pollen tubes in the style of tea plants after artificial pollination. The results demonstrated that following a period of six hours of pollination, the pollen began to germinate; after 24 h of pollination, a small number of pollen tubes reached the base of the style in the pollinated styles, and after 48 h of pollination, a large number of pollen tubes reached the base in the styles. Following a 72 h period of pollination, a few pollen tubes reached the ovary and entered the ovules [16]. The determination of the sampling time was based on this information. Compared to the FNL group, the FNH group has a greater number of pollen tube growth in each time period (Figure 2a). Subsequently, the ovary samples of EW and CM parent plants (EW-2d, EW-7d, CM-2d, and CM-7d) after pollination, as well as the ovary samples of 20 offspring with extreme traits selected from the mixed pool (FNL: S-2d and S-7d, FNH: D-2d, and D-7d), were collected, as shown in Figure 2b–e: the growth status of the offspring after pollination in the mixed pool. Figure 3f showed the normal development of the pistil (FNH) and the undeveloped pistil (FNL).

Ovary samples collected were subjected to transcriptome sequencing, resulting in EW-2d: 46.8; EW-7d: 53.2; CM-2d: 46.4; CM-7d: 47.6; D-2d: 123.3; D-7d: 92.5; S-2d: 112.4; and S-7d: 104.8 million clean reads, with Q20 values all above 97.49% and Q30 averages above 93.28, indicating a low sequencing error rate and high reliability. The clean reads were compared to the ‘Tieguanyin’ reference genome, with comparison rates generally above 85% (Table S6).

#### 3.3.2. Differential Expression Analysis and Functional Annotation

PCA is used to verify the difference between samples and the repeatability of sampling. The results show a clear separation between groups and aggregation within groups (Figure 3a), indicating that the data exhibits biological repeatability. The number of DEGs screened from each sample group is shown in Figure 3b. The EW-7d vs. EW-2d group identified the most DEGs, with a total of 7823, of which 4826 were upregulated and 2997 were downregulated. The mixed pool group D-2d vs. S-2d identified 2063, with 1140 downregulated and 923 upregulated. D-7d vs. D-2d identified 4477, with 2281 downregulated and 2196 upregulated. D-7d vs. S-7d identified 2598, with 1251 downregulated and 1347 upregulated. S-7d vs. S-2d identified 1407 genes, with 722 downregulated and 685 upregulated. As illustrated in Figure 3c, the differential expression gene clustering heatmap of the parent and mixed pool reveals that the significantly differentially expressed genes (DEGs) are mostly concentrated between EW and CM, and there are relatively fewer differentially expressed genes between the two mixed pools, which may be due to the similarity in genetic background between the mixed pools.

KEGG (Kyoto Encyclopedia of Genes and Genome, Kyoto Encyclopedia of Genes and Genomes) annotation and enrichment analysis were performed on the differentially expressed genes identified between groups. The KEGG enrichment results of DEGs among the EW, CM parent, and offspring ovary samples are shown in Figure 3d–k. In the eight comparison groups, the differentially expressed genes were mainly enriched in pathways such as “Metabolic pathways”, “Biosynthesis of secondary metabolites”, “Plant-pathogen interaction”, “Plant hormone signal transduction”, and “MAPK signaling pathway-plant”.

#### 3.3.3. BSR-Seq and Candidate Gene Screening

**Figure 4:**
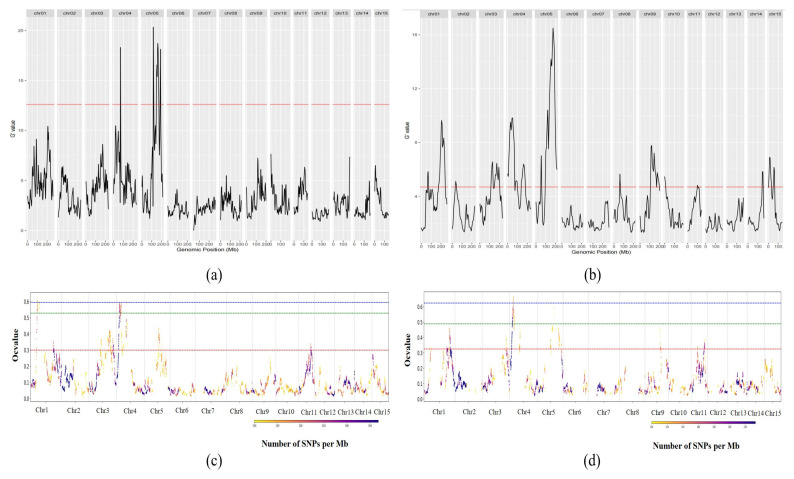
The QTL identified by G′ value and OcBSA. In (**a**), the red line represents the q = 0.05 threshold line; in (**b**), the red line represents the q = 0.01 threshold line. (**a**) G′ value localization map of extreme mixing between parents and offspring 2 days after pollination; (**b**) G′ value localization map of extreme mixing between parents and offspring 7 days after pollination. In (**c**) the red, green, and blue dashed lines in the figure represent the threshold lines for *p* = 0.05, 0.01, and 0.001, respectively. (**c**) OcBSA localization map of parents and offspring extreme mixed pools 2 days after pollination; (**d**) OcBSA localization map of parents and offspring extreme mixed pools 7 days after pollination.

Based on the G′ value and OcBSA algorithm, we mined the association intervals related to the fruit number traits of tea plants. We conducted localization analysis using the ovary transcriptome sequencing data of parents and extreme mixed pools at 2d and 7d after pollination, respectively. In the G′ value algorithm, a threshold of q = 0.01 (or 0.05) was used to screen significant intervals. The main differential candidate regions were located between 94612941 and 213433720 (118.8 Mb) on chr5 (Figure 4a,b, Tables S7 and S8). The OcBSA algorithm locates the interval between 149400000 and 181800000 (32.4 Mb) and has also locked the firmness trait-associated interval to this region (Figure 4c,d). The localization results of these two algorithms coincide with the QTL mapping and are determined to be the same QTL.

#### 3.3.4. Candidate Gene qRT-PCR Validation

**Figure 5:**
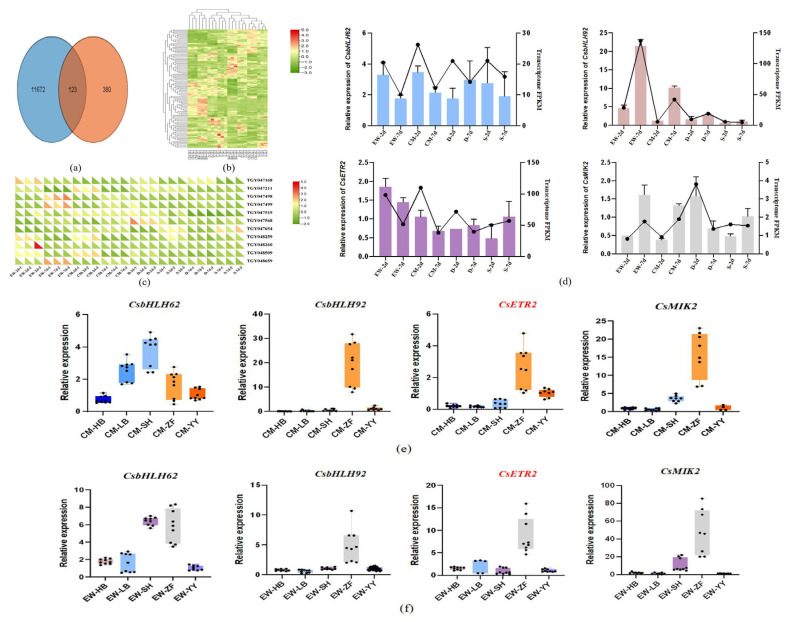
Analysis chart of differentially expressed genes, different expression genes qRT PCR validation and qRT-PCR quantitative analysis. (**a**) Venn diagram comparing the number of genes in the QTL mapping interval with the number of genes from transcriptome sequencing (**b**) heatmap of 123 genes with identical IDs (**c**) heatmap of 11 genes related to plant growth and development clustering. (**d**) The bar chart presents the qRT-PCR results using S-7d as the control, corresponding to the left vertical axis; the line chart displays the FPKM values of genes from transcriptome measurements, corresponding to the right vertical axis. (**e**,**f**) The bar chart shows the qRT-PCR results using CM-YY and EW-YY as controls. CM: ‘Chuanmu 217’, EW: ‘Emei Wenchun’, HB (hb): bud stage, LB (lb): white bud stage, SH (sh): full bloom stage, ZF (zf): ovary, YY (yy): axillary buds.

As shown in Figure 5, the chromosome interval range of the three localizations was utilized for the screening of differentially expressed genes. Within the confidence interval of the localized QTL, a total of 380 genes were predicted. These are associated with the pooled sequencing data (11,672 genes), yielding 123 genes with identical IDs (Figure 5a,b). Among them, 11 genes related to plant growth and development were discovered (Figure 5c), and ultimately four potentially key genes were filtered out: *CsbHLH62* (TGY047211), *CsbHLH92* (TGY048659), *CsETR2* (TGY048509), and *CsMIK2* (TGY047568).

To verify the reliability of the transcriptome data, qRT-PCR validation was performed on the differentially expressed genes among the selected four candidate genes based on the localization results, as shown in Figure 5d. Compared with the S-7d group, *CsbHLH62* showed upregulation in the EW-2d, CM-2d, CM-7d, D-7d, and S-2d groups, but downregulation in D-2d. *CsbHLH92* was mainly upregulated across EW-2d, EW-7d, CM-2d, CM-7d, D-2d, and D-7d groups, except for downregulation in S-2d. *CsETR2* was upregulated in EW-2d and EW-7d, downregulated in CM-7d, D-2d, D-7d, and S-2d, and stable in CM-2d. *CsMIK2* displayed upregulation in the EW-7d, CM-7d, and D-2d, alongside downregulation in EW-2d, CM-2d, D-7d, and S-2d groups. The qPCR results are significantly correlated with RNA-seq data, thus suggesting that reliable RNA-seq data were obtained from the samples.

#### 3.3.5. Candidate Gene Quantitative Analysis by qRT-PCR in Various Tissues of Tea Plants

Quantitative analysis of gene expression in various tissues of the tea plant was conducted using qRT-PCR for the four key candidate genes that were filtered. A notable tissue-specific expression pattern was observed for the selected genes in Figure 5e,f. CM-YY and EW-YY were used as reference controls. In CM, *CsbHLH92* (TGY048659), *CsETR2* (TGY048509) and *CsMIK2* (TGY047568) have the highest relative expression levels in the ovary (ZF); *CsbHLH62* (TGY047211) have the highest relative expression levels in the full bloom stage (SH). In EW, the relative expression levels of *CsbHLH62* (TGY047211), *CsbHLH92* (TGY048659), *CsETR2* (TGY048509), and *CsMIK2* (TGY047568) are the highest in the ovary. In our experiments, we found that *CsMIK2* (TGY047568) is basically not expressed in the axillary buds (YY) of EW.

#### 3.3.6. Overexpression of *CsETR2* Affects the Growth, Development, and Seed Setting of *Arabidopsis thaliana*

**Figure 6:**
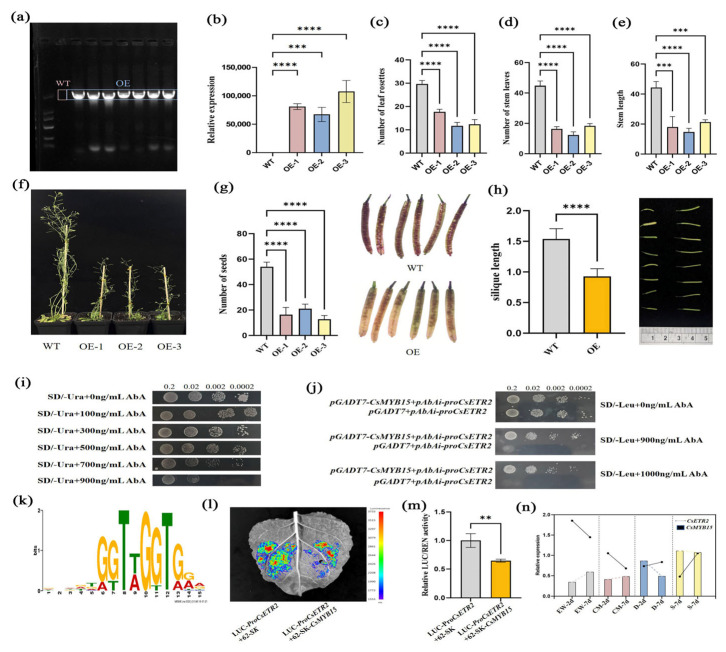
The phenotypic characteristics of overexpressing *Arabidopsis thaliana* and *CsMYB15* are able to repress *CsETR2* transcription. (**a**) Positive lines of *Arabidopsis thaliana* were identified by PCR. (**b**) Positive lines of *Arabidopsis thaliana* were analyzed by q-PCR. (**c**–**h**) Growth indexes and phenotype of WT and OE. (**i**,**j**) Yeast one-hybrid experiments showed that *CsMYB15* was able to bind the promoter of *CsETR2*. (**k**) Transcription factor binding sites. (**l**) Luciferase complementation imaging. (**m**) Quantitative analysis of luciferase. (**n**) Expression trends of *CsERT2* and *CsMYB15* in different ovary samples. “**” represents significance at *p* < 0.01, “***” represents significance at *p* < 0.001, and “****” represents significance at *p* < 0.0001.

The growth and development of plants are regulated by multiple factors, among which ethylene is one. However, the regulatory mechanism of ethylene in the fruiting of the tea plant remains unclear. Therefore, we used *Arabidopsis thaliana* as a model system to investigate the potential role of *CsETR2* in vegetative and reproductive growth. In the T_2_ generation of overexpressed *Arabidopsis thaliana*, we identified positive seedlings (Figure 6a). Compared with wild-type *Arabidopsis thaliana*, the expression level of *CsETR2* was tens of thousands of times higher (Figure 6b). The plant height of *CsETR2*-overexpressing plants was weaker than that of the wild type, with reduced effective panicles and seed setting rate (Figure 6c–f). Moreover, the silique length and seed number were lower than those of wild-type *Arabidopsis thaliana* (Figure 6g,h). These findings demonstrate its role in regulating plant fruitfulness.

#### 3.3.7. CsMYB15 Was Able to Bind the Promoter of *CsETR2*

In order to further explore the molecular mechanism by which *CsETR2* regulates fruit development in tea plants, through yeast one-hybrid experiments, it was proved that under the inhibitory concentration of 900 ng/mL AbA, the experimental group grew normally, while the control group could not grow, indicating that *CsMYB15* had specific binding ability to the promoter region of *CsETR2* (Figure 6i,j), and its binding site was shown in Figure 6k. The subsequent dual luciferase detection showed that *CsMYB15* represses the transcription of *CsETR2* by binding to its promoter. The expression level of LUC in the control group was significantly higher than that in the experimental group, so *CsMYB15* played an inhibitory role (Figure 6l,m). The quantitative fluorescence results showed that *CsETR2* and *CsMYB15* showed opposite expression patterns in the ovaries of parents and offspring (Figure 6n). Taken together, these findings suggest that *CsMYB15* represses the transcription of *CsETR2* by interacting with the promoter.

## 4. Discussion

### 4.1. Variation and Genetic Characteristics of Tea Plant Fruit Number

The flowering and fruiting of tea plants are important agronomic traits, classified as quantitative traits. These traits are controlled by multiple genes, with interactions among genes and between genes and the environment, making their genetic background extremely complex and highly variable. For hybrid breeding and genetic pattern studies, a greater number of flowers and fruits, especially a higher fruit set rate, is more advantageous. Conversely, tea plant materials that bear fewer fruits or no fruits at all are favored for the production of tea. This phenomenon is the basis of biological genetic diversity and is also key to selecting superior traits in hybrid breeding. During the process of hybrid breeding, we discovered that both the female parent EW and the male parent CM possess normal fruit number ability. However, among the 206 hybrid offspring, there was a significant variation in fruit number per plant and trait segregation observed, with single-plant yields ranging from complete sterility (0 fruits) to exceptionally high fertility (1612 fruits). Based on the records of fruit number from 2021 to 2023, the coefficients of variation for fruit number among populations were 116.69%, 187.22%, and 112.37%, respectively, indicating a relatively large potential for variation in fruit number traits. It was reported that the fruiting variation coefficients in Shengzhou and Hangzhou over two years were 200%, 159%, and 347%, and 156%, respectively, with significant variation also observed in the offspring population [4].

The reasons for the large variation coefficient in the tea plant offspring population may be multifaceted. The factors to be considered include inbreeding incompatibility, high heterozygosity of the tea genome, and pollination environment and mode. The self-incompatibility mechanism of tea plants may affect their reproductive methods, leading to higher genetic diversity in their offspring. The self-incompatibility mechanism may involve different types of SI, such as gametophytic self-incompatibility (GSI) or sporophytic self-incompatibility (LSI). Some literature suggests that tea plants may possess LSI, meaning that the pollen tube is only inhibited after entering the ovary, which could lead to different fertilization success rates among different offspring. The molecular mechanism underlying this phenomenon was complicated. For instance, genes related to reactive oxygen species (ROS) metabolism play a significant role in self-incompatibility, with the significant difference in expression patterns between self-pollination and cross-pollination [17]. Furthermore, calcium-dependent protein kinases (CDPKs), S-proteins, PR-1 proteins, and others also function in self-incompatibility. All of these genes regulated the growth of pollen tubes and the fertilization process, further inhibiting self-pollination [18]. The high heterozygosity of the tea plant genome may also lead to a diverse combination of genes after genetic recombination in the offspring [19]. Highly heterozygous parents’ recessive harmful mutations homozygose in offspring, leading to partial individual embryo abortion or reduced seed set rate, resulting in significant differences in fruit number [20]. Furthermore, the variation within the full-sibling population may be associated with genetic linkage disequilibrium and epigenetic regulation (Linkage Disequilibrium, LD). DNA methylation, such as H3K27Ac modification of the CsMADS27 gene, and miRNA (such as the miR172-AP2 pathway regulating bud dormancy) may dynamically regulate the expression of reproduction-related genes, increasing phenotypic variation [21].

### 4.2. The QTL Mapping of Fruit Number Traits

The analysis and confirmation of potential regulatory genes for important traits of economic crops through QTL mapping will accelerate the process of genetic improvement. In the QTL mapping for traits such as spike length and seed characteristics in wheat, a total of 68 QTLs were identified, among which 12 QTLs could be stably detected across different environments, which can be used for wheat improvement [22]. In maize, an F2 and F2:3 population was constructed using the non-multi-ear inbred line Mo17 and a multi-ear material, LAN404, from a local variety as parents. A QTL, *qEN7*, affecting ear number was identified, which can explain 10.7% to 11.9% of the phenotypic variation. The allele from the local variety can increase the ear number by approximately 1. Based on this, through fine mapping, the *qEN7* interval was narrowed down to 0.56 Mb, and eight candidate genes related to ear number were identified in this region, which will provide an important reference for corn ear number improvement [23].

The above crops, such as wheat and corn, generally construct F_2_ populations or recombinant inbred lines for genetic mapping. However, the development of F_2_ populations in tea plants presents a significant challenge, primarily due to their long growth cycles, self-incompatibility, and low seed-setting rates. However, due to their highly heterozygous genomes, genetic maps can be constructed using F_1_ populations based on the “pseudo-testcross” strategy (in the pseudo-testcross configuration, markers are present in one parent and absent in the other or vice versa and are expected to segregate 1:1 in the F_1_ generation) [24]. Research on the genetic map of tea plants started relatively late, and there are currently few studies on QTL mapping related to tea plant fruit number traits both domestically and internationally. Wei et al. utilized the ‘Longjing 43’ × ‘Baihaozao’ hybrid F_1_ population to map the fruit number trait, identifying four overlapping QTLs that were mapped to the LG1, 2, 5, and 15 chromosomes of the reference tea genome ‘shuchazao’ [2]. Wang et al. utilized the F_1_ population (*n* = 324) constructed from ‘Longjing 43’ × ‘Baihao Zao’ and built a high-density genetic map to locate genes associated with fruit number rate. A total of six QTLs related to fruit number traits were mapped, accurately positioned on LG01, LG02, LG03, LG04, LG05, LG06, LG07, and LG11 of ‘Shuchazao’. The most stable QTL was located on LG03 [4]. The stable QTL interval identified in this study was located on Chr5 of ‘Tieguanyin’, and the QTL was locked in a 32.4 Mb interval.

### 4.3. The Application of BSR-Seq in Genetic Mapping of Tea Plants

At present, BSR-seq (Bulked Segregant RNA-Seq) is a novel gene localization strategy combining BSA and transcriptome sequencing (RNA-seq) [25]. This method directly correlates gene expression differences through transcriptome sequencing, which significantly improves the efficiency of candidate gene screening. BSR-seq research on the fruit number trait of economic crops is relatively scarce. Zhao et al. utilized 188 core peanut germplasm samples for identification, selecting two peanut materials with significant differences in pod size/weight to construct F_2_ populations. The BSA-Seq technology generated 288.58 Gb of raw data. By employing 285,914 high-quality SNPs and 70,759 InDel markers, the researchers localized genes controlling pod size within a 1.17 Mb region on chromosome 07 [26]. Ma et al. cloned the CNS1 gene from a maize seedling developmental defect mutant by using BSR-seq technology. The CNS1 gene encodes the P subtype of PPR protein, which is crucial for maintaining the function of mitochondrial complex III and ensuring the mitochondrial biogenesis and seed development in maize [27]. In this study, BSR-seq technology was utilized in conjunction with the G value algorithm and the OcBSA algorithm to narrow down the interval to chromosome 5 (32.4 Mb), which aligns with the QTL results.

### 4.4. CsBHLH92 May Be Involved in Regulating Tea Plant Fruit Set

The key molecular mechanisms of plant fruit number traits regulation involve multiple aspects, including hormone signal transduction, gene expression regulation, metabolic pathways, and the comprehensive impact of environmental factors. In this study, QTL mapping and BSR-seq combined with gene function annotation and literature analysis identified the candidate gene *CsbHLH92* (TGY048659) that may be related to fruit set.

Transcription factors are important regulatory proteins in the life process of plants, playing a significant role in plant growth and development. The bHLH (basic helix-loop-helix) transcription factor family is one of the largest families of plant transcription factors. Members of this family bind to the promoters of target genes through a conserved helix-loop-helix domain and are involved in regulating numerous plant growth and developmental processes, such as adaptation to biotic and abiotic stress, flower development, and seed maturation and germination. The functional analysis of these family members holds great potential value for crop breeding [28,29,30]. According to research findings, the *bHLH92* homologous gene plays a key role in the yield of rice. In the latest study, Lu et al. revealed a new pathway for the antagonistic regulation of rice brassinosteroid signaling and plant architecture grain shape formation by the ILIs-OsAIFs-OsbHLH92 module. This precise multiple antagonistic mechanism achieves precise regulation of rice BR signaling and plant architecture grain shape [31]. Teng et al. demonstrated through positional cloning, *CRISPR/Cas9*, and overexpression experiments that the overexpression of *OsbHLH92* is the main cause of increased leaf angle, revealing the role of the rice bHLH family member *OsbHLH92* in enhancing the photosynthetic efficiency and yield of rice [32]. The relative expression of *CsbHLH92* was found to be the highest in the ovary tissue of tea plants in this study. It is speculated that this gene may be involved in the development of the ovary, but the specific regulatory mechanism remains to be studied.

### 4.5. Functional Characterization of CsETR2 in Tea Plants

Ethylene (ETH) is a gaseous plant hormone that affects plant growth and development, such as breaking seed dormancy, promoting flowering, fruit ripening, etc. [33]. When plants are pollinated, ethylene production is induced to accelerate the ovary’s transformation into a fruit. Ethylene synthesis genes and signaling transduction genes show higher expression levels in unpollinated tomato ovaries. Notably, pollination itself can instantly boost ethylene concentrations in the pistil across various plant species, though these levels gradually decrease as fruits develop [34,35]. Ethylene induced by pollination is important for the coordination of ovary growth and flower senescence. The results of the tomato ovary transcriptome study showed that the expression levels of many ethylene-related genes changed during the transition from flower to fruit [34,35,36]. These genes include both those involved in their synthesis and those involved in the ethylene signaling pathway. The overexpression of the SlTPR1 gene in ethylene can induce parthenogeny and also promote leaf and flower senescence, fruit ripening, and organ abscission [37]. The study by Ogawara et al. indicates that ethylene is involved in the transition from asexual reproduction to reproductive growth in Arabidopsis, and the ethylene receptor mutant ETR1-1, which has a functional gain mutation, exhibits late flowering [38]. Wuriyanghan et al. discovered that the ethylene receptor (ethylene receptor 2, *ETR2*) delays the transformation of rice flowering, affects starch accumulation, and in overexpressing plants, the number of effective panicles and seed setting rate are reduced [39]. In this process, ethylene is also regulated by many transcription factors.

In this study, the yeast one-hybrid assay identified that *CsMYB15* can bind to the *CsETR2* promoter and affect its transcriptional activity. In the dual-luciferase assay, *CsMYB15* reduced the activity of the *CsETR2* promoter by 35%. These results indicated that *CsMYB15* can negatively regulate the expression of the *CsETR2* gene. RT-qPCR analysis confirmed the successful overexpression of *CsETR2* in transgenic Arabidopsis thaliana lines. The relative expression levels of *CsETR2* in these transgenic lines were elevated by several orders of magnitude, reaching levels tens of thousands of times higher than those observed in the wild-type (WT). The plant height of CsETR2-overexpressing plants was weaker than that of the wild type, with reduced effective panicles and seed setting rate. Relative to the wild type, the transgenic lines exhibited the number of effective panicles decreased by 72.5%, the seed setting rate dropped by 67.7%, and the silique length shortened by 38%. The reproductive performance of the transgenic plants was significantly lower than that of the wild-type plants. These findings indicate that overexpression of *CsETR2* exerts a certain inhibitory effect on the growth, development, and seed setting of *Arabidopsis thaliana*, and this gene may play a similar role in tea plants.

## 5. Conclusions

In conclusion, a stable QTL for fruit number was mapped to chromosome 5 in both 2021 and 2023, as revealed by linkage analysis. We performed transcriptome analysis coupled with the OcBSA and G′ value algorithms, which identified three significant genomic intervals. Notably, the interval on chromosome 5 exhibited a high degree of overlap with the previously mapped *qFN5*, and the final QTL was locked at the 32.4 Mb region. Analysis of expression dynamics combined with functional genomics data revealed a prominent candidate gene, *CsETR2* (TGY048509), which encodes an ethylene receptor protein. When *CsETR2* was overexpressed in *Arabidopsis*, transgenic lines exhibited significantly decreased reproductive performance relative to wild-type plants these findings demonstrate its role in regulating plant fruitfulness. Furthermore, yeast one-hybrid and dual-luciferase assays confirmed that *CsMYB15* (TGY110225) represses the transcription of *CsETR2* by binding to its promoter. In summary, this study establishes the CsMYB15-CsETR2 module as a core determinant of tea plant fruiting, providing a pivotal genetic basis of fruiting traits in tea plants and for accelerating molecular breeding programs.

## Data Availability

Data will be made available on request.

## Supplementary Materials

The following supporting information can be downloaded at: https://www.mdpi.com/article/10.3390/plants14192963/s1, Table S1: Related PCR primer sequences; Table S2: fruit number trait in the EW × CM217 F1 population in 3 different years; Table S3: Phenotypic traits statistics of F1 progeny and their parents; Table S4: Marker positions on the genetic and physical maps; Table S5: QTL mapping results for F1 progeny groups FN2021 and FN2023; Table S6: Sequencing Data Statistics; Table S7: Chromosome interval location information (Figure 4a); Table S8: Chromosome interval location information (Figure 4b).
