# Supplementary material for: Integrating Genetic Mapping and BSR-Seq Analysis to Identify Candidate Genes Controlling Fruitfulness in Camellia sinensis
Source: Plants (Basel). 2025 Sep 24;14(19):2963. doi: 10.3390/plants14192963 (PMC12526348; doi:10.3390/plants14192963)
Supplement: Supplementary file 1 [file plants-14-02963-s001.zip › plants-3867279-supplementary.pdf]

Supplementary table. S1 Related PCR primer sequences

Primer name	Forward Primers	Reverse Primers	Product length/bp
bHLH92-qPCR（qRT-PCR）	ATCAGAGCGGTTTTGTTAGATG	GATTCACTGGTAAAGCCTCCT	156
bHLH62-qPCR（qRT-PCR）	AGTGAGGACAACACAATATTACC	GGATCGCTCTGATCCATTGAA	146
ETR2-qPCR（qRT-PCR）	TGCAGTGGCCTATTTCTCTATC	GGTGAGGGCTAGCATTAGCT	178
MIK2-qPCR（qRT-PCR）	ATCAGCTAGCAAAGCCCAATC	GTTGCATGCTATGCCACTCCA	166
CsGAPDH （qRT-PCR）	TTGGCATCGTTGAGGGTCT	CAGTGGGAACACGGAAAGC	210
AtActin2（qRT-PCR）	GACCTTGCTGGACGTGACCTTAC	GTAGTCAACAGCAACAAAGGAGAGC	135
MYB15-qPCR（qRT-PCR）	TCCATGGCCATCCCAATTGG	CTGACCATCTATTTCCCAACT	180
CsETR2-35S-EGFP	ACGAACGATAGCCATGGTACCATGTTA AAGACTTTAGCATATGGGC	GCCTGCGGCCGCGCCGGATCCCACAACT TTGTGTGCCATTTGCA	2292
CsETR2- pGreenII 0800-Luc	ACTCACTATAGGGCGAATTGGGTACCA ATAATGATATCCAAGTTATTCTC	GCTGCAGGAATTCGATATCAAGCTTTTC ATCCAATGGATAAATTCTTCA	1938
MYB15-62sk	GGCCGCTCTAGAACTAGTGGATCCAT GGTGAGAGCTCCATGCTG	GAGGTCGACGGTATCGATAAGCTTCTAA AACTCGGTCAAATTTGGT	771
PAbAi- *proCsETR2*	AAATGATGAATTGAAAAGCTTCTGAGG GTTGGTGGGCTGAGGGTTGGTGGG	CATACAGAGCACATGCCTCGAGCCCACC AACCCTCAGCCCACCAACCCTCAG	1938
pGADT7-*MYB15*	ACGTACCAGATTACGCTCATATGATGG TGAGAGCTCCATGCTG	CTGCAGCTCGAGCTCGATGGATCCCTAA AACTCGGTCAAATTTGGT	771

Supplementary Table S2. fruit number trait in the EW × CM217 F1 population in 3 different years

ID（F1）	2021	2022	2023
103	36	0	28
104	46	0	114
106	0	3	0
108	*	*	*
112	122	0	87
116	99	0	2
117	56	0	98
123	236	0	64
124	249	0	7
125	69	1	48
126	60	0	21
127	2	0	20
129	56	0	57
130	1	0	23
131	140	0	18
136	32	7	155
145	232	1	39

146	19	0	78
202	34	1	94
206	7	0	111
210	12	0	67
212	135	0	85
217	114	1	77
218	138	0	81
219	0	0	59
226	52	0	101
228	30	0	13
229	7	0	46
230	106	9	141
234	0	0	2
239	0	26	113
240	65	0	148
241	145	9	220
244	73	3	164
246	*	*	*
250	120	0	155
252	280	2	524
305	0	0	0
310	0	0	0
317	110	11	483
318	0	0	0
319	61	0	55
326	84	0	0
328	49	5	385
332	0	0	0
334	*	*	*
337	*	*	*
338	111	0	44
339	23	0	177
343	104	0	17
403	0	0	0
404	0	0	0
406	38	6	42
407	136	0	151
411	59	0	12
413	27	2	212
415	16	6	212
416	23	0	93
417	95	17	285
418	35	21	132

420	33	0	134
421	10	2	199
423	44	34	219
425	7	1	29
428	133	12	191
429	229	0	189
431	73	1	79
432	235	0	89
435	150	0	25
437	81	1	88
439	*	*	*
440	75	0	626
502	105	0	62
503	0	0	0
506	112	1	63
507	127	1	14
509	92	5	120
511	9	7	9
513	66	6	165
517	0	0	0
519	0	0	0
521	37	35	246
525	85	0	148
526	0	0	0
527	18	7	120
530	202	0	335
532	92	2	286
538	0	0	0
541	238	4	195
601	378	3	123
602	142	12	314
606	123	5	224
608	119	33	424
609	57	16	213
614	233	5	402
621		16	221
625	89	61	80
630	112	0	81
631	265	1	372
633	111	14	144
634	71	0	20
635	327	2	173
639	305	6	620

640	*	*	*
641	137	3	142
701	0	3	8
702	39	8	202
704	58	3	357
705	77	24	245
707	0	0	0
713	130	1	254
715	44	15	53
718	169	48	180
720	109	6	309
722	68	4	367
727	0	0	0
730	627	2	510
736	70	55	205
738	*	*	*
801	22	1	49
803	0	0	0
804	282	11	438
807	0	0	0
808	34	47	14
810	277	19	416
811	3	14	0
813	0	0	0
822	0	10	314
825	191	0	204
826	38	13	421
830	0	0	5
833	0	0	0
836	66	9	276
837	55	162	141
839	178	15	1612
902	63	32	289
904	92	45	305
906	264	43	323
911	18	4	238
912	328	54	327
917	0	0	0
923	51	22	286
1002	71	21	371
1003	74	1	123
1007	141	50	519
1038	0	*	*

1102	0	0	0
1105	0	0	0
1106	19	61	140
1107	0	0	0
1108	23	12	162
1113	318	34	602
1116	4	56	436
1119	0	0	0
1121	12	38	296
1123	36	1	221
1203	62	43	116
1213	8	41	242
1216	27	82	564
1304	26	52	188
1307	202	70	280
1308	136	32	13
1309	189	53	200
1312	154	7	366
1313	6	32	205
1318	21	8	202
1321	70	1	1
1324	*	*	*
1325	114	8	253
1331	153	178	85
1332	*	*	*
1337	*	*	*
1340	34	23	552
1406	185	104	212
1421	177	11	311
1429	0	0	0
1435	36	27	140
1437	13	1	180
1440	378	6	301
1442	135	105	684
1501	1	7	7
1502	82	31	71
1507	8	0	3
1511	0	0	0
1512	2	21	239
1534	18	52	470
1536	32	0	374
1538	1	35	0
1541	121	8	229

1544	190	5	439
1548	213	10	259
1549	42	11	242
1551	121	9	554
1552	0	0	0
1607	4	0	2
1608	10	14	38
1609	15	0	65
1618	1	0	50
1619	0	0	0
1620	11	0	35
1621	0	0	122
1623	0	11	231
1624	9	0	0
1626	9	7	84
1629	78	16	81
1631	44	42	161
1632	1	5	28
1644	0	0	0
1650	189	0	120
1652	129	1	405
1656	0	0	0
1657	30	89	156
1658	155	58	401
1659	256	58	645
1661	134	58	568
1663	30	0	329
1664	0	0	0

Supplementary table. S3 Phenotypic traits statistics of F1 progeny and their parents

Year	Minimum value	Maximum value	Average value	Standard deviation	Skewness	Kurtosis	Coefficient of Variation
2021	0	627	80.48	93.91	1.91	5.66	116.69%
2022	0	178	13.61	25.48	3.30	14.44	187.22%
2023	0	1612	171.19	192.37	2.66	14.57	112.37%

Supplementary Table S4. Marker positions on the genetic and physical maps

Linkage group / chromosome	Genetic position	Marker ID	Physical positions
Chr1	0	Marker331	1645945
Chr1	0	Marker332	1646002
Chr1	0	Marker48	161730

Chr1	0.93	Marker251	1186272
Chr1	0.93	Marker443	2125455
Chr1	0.93	Marker624	3945065
Chr1	0.93	Marker74	228949
Chr1	0.93	Marker76	229023
Chr1	1.281	Marker479	2258097
Chr1	1.281	Marker726	4785867
Chr1	1.613	Marker218	979616
Chr1	1.613	Marker381	1853743
Chr1	1.631	Marker779	4998822
Chr1	1.981	Marker966	6638716
Chr1	1.981	Marker971	6677303
Chr1	2.984	Marker654	4216661
Chr1	3.666	Marker811	5361422
Chr1	4.349	Marker1040	7454246
Chr1	4.349	Marker1206	8365413
Chr1	4.349	Marker1431	10656558
Chr1	4.349	Marker876	6078916
Chr1	6.415	Marker1535	11778178
Chr1	6.415	Marker1637	12962848
Chr1	6.415	Marker1679	13109871
Chr1	6.415	Marker1680	13110141
Chr1	6.415	Marker2102	17384602
Chr1	7.036	Marker1233	8410583
Chr1	8.609	Marker1669	13090929
Chr1	8.609	Marker1796	15118380
Chr1	8.609	Marker1935	16181558
Chr1	8.609	Marker1960	16511162
Chr1	8.917	Marker2323	19482260
Chr1	10.17	Marker2374	19686426
Chr1	10.17	Marker2491	21250863
Chr1	10.17	Marker2545	21510657
Chr1	11.109	Marker2243	18815440
Chr1	11.109	Marker2261	19018546
Chr1	11.109	Marker2379	19727137
Chr1	12.361	Marker2422	20329888
Chr1	12.669	Marker2497	21315942
Chr1	12.669	Marker2506	21353696
Chr1	12.978	Marker2567	22239473
Chr1	13.913	Marker2689	22797429
Chr1	13.913	Marker2966	25600600
Chr1	14.21	Marker2977	25626547
Chr1	14.21	Marker2999	25823666

Chr1	14.959	Marker3002	25842340
Chr1	15.11	Marker3078	26324607
Chr1	15.708	Marker3731	30630303
Chr1	15.708	Marker3761	30669734
Chr1	16.005	Marker3277	27351997
Chr1	16.005	Marker3448	28967605
Chr1	16.749	Marker3278	27352007
Chr1	17.246	Marker3792	30684110
Chr1	17.493	Marker3766	30670035
Chr1	17.863	Marker3862	31131314
Chr1	17.863	Marker4077	33053498
Chr1	19.071	Marker3878	31370072
Chr1	19.071	Marker3992	32492430
Chr1	20.28	Marker4051	32827464
Chr1	20.28	Marker4121	33280574
Chr1	20.28	Marker4452	37125204
Chr1	20.28	Marker4459	37199716
Chr1	21.488	Marker4471	37223149
Chr1	21.488	Marker4476	37264448
Chr1	21.488	Marker4491	37581709
Chr1	21.488	Marker4829	39949607
Chr1	21.488	Marker4859	40068156
Chr1	21.488	Marker4889	40258974
Chr1	21.719	Marker4875	40095272
Chr1	22.536	Marker4928	40611747
Chr1	22.806	Marker5103	42184212
Chr1	23.348	Marker5131	42333611
Chr1	24.049	Marker5321	43616282
Chr1	24.049	Marker5678	45567356
Chr1	24.75	Marker5001	41223348
Chr1	24.75	Marker5696	45638979
Chr1	24.75	Marker5722	45754427
Chr1	24.75	Marker5792	46454501
Chr1	24.75	Marker5805	46456559
Chr1	24.75	Marker6150	48153282
Chr1	27.941	Marker5826	46748000
Chr1	28.88	Marker5831	46748309
Chr1	28.88	Marker5840	46847983
Chr1	28.88	Marker6826	51399504
Chr1	29.819	Marker5842	46848301
Chr1	29.819	Marker5944	47210932
Chr1	29.819	Marker6181	48206079
Chr1	29.819	Marker6284	48937725

Chr1	29.819	Marker6364	49411831
Chr1	29.819	Marker6599	50093625
Chr1	29.819	Marker6613	50263247
Chr1	30.281	Marker6962	52349044
Chr1	30.742	Marker7204	54226467
Chr1	30.742	Marker7220	54272243
Chr1	31.226	Marker6610	50212717
Chr1	31.226	Marker6703	50894684
Chr1	31.226	Marker7194	54155392
Chr1	31.226	Marker7196	54160060
Chr1	31.226	Marker7200	54191924
Chr1	32.159	Marker6832	51465384
Chr1	32.159	Marker7492	56452683
Chr1	32.159	Marker7551	56534969
Chr1	32.159	Marker7635	56837491
Chr1	32.159	Marker7663	57037684
Chr1	33.093	Marker7320	55737404
Chr1	33.093	Marker7398	55926501
Chr1	33.093	Marker7640	56839650
Chr1	34.033	Marker7752	57435978
Chr1	34.502	Marker7754	57436067
Chr1	34.97	Marker8157	60812517
Chr1	34.97	Marker8161	60852963
Chr1	35.438	Marker8445	62935456
Chr1	35.906	Marker8133	59962034
Chr1	35.906	Marker8196	61468910
Chr1	35.906	Marker8259	61640236
Chr1	35.906	Marker8313	61854272
Chr1	35.906	Marker8401	62653140
Chr1	35.906	Marker8539	63765598
Chr1	35.906	Marker8655	64092114
Chr1	35.906	Marker8658	64132544
Chr1	35.906	Marker8681	64355005
Chr1	37.475	Marker9247	67849079
Chr1	37.475	Marker9379	68461391
Chr1	37.475	Marker9380	68483561
Chr1	37.927	Marker10730	73728791
Chr1	37.927	Marker10731	73729084
Chr1	37.927	Marker10737	73777062
Chr1	37.927	Marker10747	73818382
Chr1	37.927	Marker10866	74209617
Chr1	37.927	Marker10871	74216786
Chr1	37.927	Marker10872	74226692

Chr1	37.927	Marker10924	74545033
Chr1	37.927	Marker10932	74558375
Chr1	37.927	Marker11124	75667885
Chr1	37.927	Marker9533	69228757
Chr1	38.256	Marker11149	75799086
Chr1	38.256	Marker9016	66190050
Chr1	38.256	Marker9123	66842527
Chr1	38.256	Marker9374	68404491
Chr1	38.256	Marker9489	69149523
Chr1	38.488	Marker9496	69168828
Chr1	38.796	Marker10501	72330624
Chr1	39.416	Marker10609	72854497
Chr1	39.416	Marker11137	75721665
Chr1	39.416	Marker9382	68490511
Chr1	41.004	Marker9059	66442461
Chr1	41.004	Marker9075	66533735
Chr1	41.004	Marker9355	68218488
Chr1	41.528	Marker11148	75798792
Chr1	41.528	Marker11618	78717623
Chr1	41.991	Marker10048	70802354
Chr1	41.991	Marker10439	72174586
Chr1	41.991	Marker10611	72855748
Chr1	41.991	Marker11194	75998386
Chr1	41.991	Marker11202	76158290
Chr1	41.991	Marker11440	77646986
Chr1	41.991	Marker11506	78167577
Chr1	41.991	Marker11551	78309551
Chr1	41.991	Marker11596	78552916
Chr1	41.991	Marker11607	78641533
Chr1	41.991	Marker12220	80937681
Chr1	41.991	Marker9840	70405707
Chr1	42.454	Marker11164	75843471
Chr1	42.454	Marker12937	86219484
Chr1	42.685	Marker11126	75668154
Chr1	42.685	Marker11333	77099813
Chr1	42.685	Marker11441	77647264
Chr1	43.15	Marker11490	78085375
Chr1	43.15	Marker11597	78553139
Chr1	43.15	Marker11603	78593287
Chr1	43.15	Marker11757	78996401
Chr1	43.15	Marker12361	82244500
Chr1	43.15	Marker12368	82281426
Chr1	43.15	Marker12692	84141032

Chr1	44.561	Marker12685	84140769
Chr1	44.561	Marker12845	85639232
Chr1	44.561	Marker12874	85842501
Chr1	45.142	Marker13037	86917163
Chr1	45.142	Marker13228	88994972
Chr1	45.432	Marker13254	89205293
Chr1	45.432	Marker13368	89751263
Chr1	45.721	Marker13199	88811818
Chr1	45.721	Marker13288	89377290
Chr1	45.721	Marker13404	89876091
Chr1	45.721	Marker13416	89911326
Chr1	45.721	Marker13424	89936920
Chr1	45.721	Marker13434	90001504
Chr1	45.721	Marker13496	90638081
Chr1	45.721	Marker13500	90668327
Chr1	45.721	Marker13631	91242054
Chr1	45.721	Marker13638	91252940
Chr1	45.721	Marker13651	91279303
Chr1	47.555	Marker14705	95227489
Chr1	47.555	Marker14895	96376658
Chr1	47.555	Marker15132	97070286
Chr1	47.555	Marker15193	97201257
Chr1	48.278	Marker14199	93390825
Chr1	48.278	Marker14226	93417173
Chr1	48.278	Marker14388	94031345
Chr1	48.353	Marker13754	91808930
Chr1	48.353	Marker13783	91870503
Chr1	48.353	Marker13784	91894876
Chr1	48.353	Marker13786	91895074
Chr1	48.353	Marker13798	91930310
Chr1	48.353	Marker13858	92101739
Chr1	49.001	Marker14166	93349637
Chr1	49.001	Marker14623	94979959
Chr1	49.001	Marker14625	94980014
Chr1	49.466	Marker12708	84475358
Chr1	49.466	Marker12839	85638958
Chr1	49.466	Marker12857	85722611
Chr1	49.466	Marker13870	92103409
Chr1	49.466	Marker14039	92742963
Chr1	49.466	Marker14553	94675723
Chr1	49.466	Marker14617	94921503
Chr1	49.466	Marker14688	95220388
Chr1	49.466	Marker14734	95439534

Chr1	49.929	Marker13245	89095518
Chr1	49.929	Marker13755	91809191
Chr1	49.929	Marker13759	91812576
Chr1	50.392	Marker13893	92212230
Chr1	50.392	Marker13946	92301583
Chr1	50.392	Marker13983	92524321
Chr1	51.231	Marker14225	93416953
Chr1	51.231	Marker14256	93519503
Chr1	51.967	Marker15195	97241805
Chr1	52.069	Marker15258	97775574
Chr1	52.486	Marker15072	96970781
Chr1	52.486	Marker15838	102288312
Chr1	52.486	Marker15848	102306445
Chr1	52.949	Marker14953	96579597
Chr1	52.949	Marker15569	100723880
Chr1	53.28	Marker15233	97471747
Chr1	53.724	Marker15385	98597209
Chr1	53.724	Marker15418	99228588
Chr1	53.724	Marker15558	100588415
Chr1	54.28	Marker15871	102319580
Chr1	54.28	Marker15878	102334753
Chr1	55.279	Marker15879	102348644
Chr1	55.279	Marker15933	102377473
Chr1	55.745	Marker16354	104843607
Chr1	55.745	Marker16729	107049604
Chr1	56.208	Marker16399	104952946
Chr1	56.208	Marker16527	105819811
Chr1	56.637	Marker15966	102611514
Chr1	56.637	Marker16150	104143550
Chr1	56.637	Marker16351	104834738
Chr1	56.637	Marker16401	104953208
Chr1	56.637	Marker16495	105535286
Chr1	56.71	Marker16530	105820052
Chr1	56.71	Marker16664	106874113
Chr1	56.71	Marker16862	107744514
Chr1	56.71	Marker17105	109654388
Chr1	57.721	Marker17106	109658642
Chr1	57.721	Marker17289	110326718
Chr1	57.721	Marker17309	110381629
Chr1	57.721	Marker17329	110428586
Chr1	57.721	Marker17373	110806624
Chr1	57.721	Marker17622	111839886
Chr1	58.377	Marker17113	109659377

Chr1	58.377	Marker17491	111366956
Chr1	58.377	Marker17600	111629460
Chr1	58.377	Marker18185	114253059
Chr1	58.377	Marker18314	114915817
Chr1	58.377	Marker18339	115206022
Chr1	58.377	Marker18361	115272645
Chr1	58.377	Marker18459	116375241
Chr1	58.731	Marker17662	112070101
Chr1	58.731	Marker18107	113967443
Chr1	58.805	Marker18583	117024691
Chr1	58.805	Marker18588	117196029
Chr1	58.805	Marker18658	117335165
Chr1	58.805	Marker18794	118787782
Chr1	58.805	Marker19448	124416276
Chr1	58.805	Marker19499	125083943
Chr1	59.234	Marker18130	114066977
Chr1	59.234	Marker18162	114149784
Chr1	59.234	Marker18181	114252813
Chr1	59.234	Marker18336	115205932
Chr1	60.414	Marker17320	110416580
Chr1	60.414	Marker18134	114067254
Chr1	60.414	Marker18268	114802427
Chr1	60.414	Marker18429	116223994
Chr1	60.414	Marker18527	116771438
Chr1	60.645	Marker17557	111535235
Chr1	60.645	Marker18530	116777823
Chr1	60.645	Marker18749	118561226
Chr1	60.645	Marker18913	120145146
Chr1	61.342	Marker18649	117328556
Chr1	61.342	Marker18911	120144869
Chr1	61.342	Marker19354	123647097
Chr1	61.342	Marker19437	124371281
Chr1	61.342	Marker19446	124415988
Chr1	61.689	Marker18845	119404796
Chr1	61.689	Marker18884	119947087
Chr1	62.739	Marker19209	122517171
Chr1	62.739	Marker19680	126598634
Chr1	63.91	Marker19528	125367150
Chr1	63.91	Marker19546	125476272
Chr1	63.91	Marker19675	126451274
Chr1	63.91	Marker20008	128129371
Chr1	63.91	Marker20233	129938162
Chr1	65.332	Marker19049	121022356

Chr1	65.332	Marker19466	124524139
Chr1	65.332	Marker19534	125384108
Chr1	65.332	Marker19921	127629739
Chr1	65.332	Marker20231	129937921
Chr1	65.332	Marker20255	130050257
Chr1	65.332	Marker20318	130403886
Chr1	65.332	Marker20328	130404154
Chr1	65.797	Marker19665	126347624
Chr1	65.797	Marker20359	131110929
Chr1	65.797	Marker20411	131677958
Chr1	66.494	Marker20459	132210053
Chr1	66.494	Marker20488	132354840
Chr1	67.426	Marker20508	132479978
Chr1	67.426	Marker20593	133111205
Chr1	67.426	Marker20602	133150107
Chr1	67.889	Marker20510	132480189
Chr1	67.889	Marker20909	135401925
Chr1	67.889	Marker20915	135415264
Chr1	67.889	Marker20923	135438933
Chr1	67.889	Marker21270	138594349
Chr1	67.889	Marker21300	138792400
Chr1	68.59	Marker21443	139515776
Chr1	68.822	Marker21843	142000488
Chr1	68.822	Marker21856	142234254
Chr1	68.822	Marker21864	142278350
Chr1	68.822	Marker21870	142289635
Chr1	69.053	Marker20666	134048983
Chr1	69.053	Marker20669	134049269
Chr1	69.053	Marker20913	135402145
Chr1	69.053	Marker21788	141738628
Chr1	69.053	Marker21918	142479696
Chr1	71.45	Marker22172	143861185
Chr1	71.45	Marker22218	144356430
Chr1	72.389	Marker22054	143195378
Chr1	72.389	Marker22227	144386501
Chr1	72.389	Marker22504	146087329
Chr1	72.389	Marker22749	148520547
Chr1	72.389	Marker22781	148759206
Chr1	72.389	Marker22801	148855451
Chr1	72.389	Marker22852	148987394
Chr1	72.707	Marker21526	140225903
Chr1	72.707	Marker21908	142378442
Chr1	72.707	Marker21928	142482677

Chr1	73.024	Marker22147	143681699
Chr1	73.024	Marker22260	144460281
Chr1	73.663	Marker23311	152359805
Chr1	73.663	Marker23356	152581828
Chr1	74.968	Marker22860	149007306
Chr1	74.968	Marker22975	149432528
Chr1	74.968	Marker23574	154176017
Chr1	74.968	Marker23607	154266989
Chr1	74.968	Marker23608	154279455
Chr1	74.968	Marker23672	154440943
Chr1	74.968	Marker23678	154441244
Chr1	74.968	Marker23703	154822742
Chr1	74.968	Marker23779	155274183
Chr1	74.968	Marker23838	156443248
Chr1	74.968	Marker24316	158484211
Chr1	74.968	Marker24514	160308107
Chr1	75.953	Marker24187	157684741
Chr1	75.953	Marker24314	158483950
Chr1	76.27	Marker24384	159027471
Chr1	76.27	Marker24428	159539088
Chr1	76.27	Marker24448	159645707
Chr1	76.909	Marker24730	161220767
Chr1	76.909	Marker24839	161608790
Chr1	77.548	Marker24521	160330446
Chr1	77.548	Marker24790	161305157
Chr1	77.548	Marker24812	161475255
Chr1	77.548	Marker24926	162525356
Chr1	77.548	Marker25086	163648281
Chr1	77.548	Marker25130	164221622
Chr1	78.487	Marker25092	163757496
Chr1	78.487	Marker25139	164222040
Chr1	78.487	Marker25544	166900131
Chr1	79.3	Marker25342	165699551
Chr1	79.574	Marker25672	168148546
Chr1	80.113	Marker25546	166932292
Chr1	80.115	Marker25590	167228198
Chr1	81.754	Marker25833	169971710
Chr1	81.754	Marker25836	170145225
Chr1	81.754	Marker25848	170296102
Chr1	81.754	Marker25861	170339774
Chr1	81.754	Marker26145	172000381
Chr1	81.754	Marker26183	172137340
Chr1	81.754	Marker26191	172161136

Chr1	82.924	Marker26004	170848519
Chr1	82.924	Marker26285	172581544
Chr1	83.272	Marker26050	170976286
Chr1	83.619	Marker26068	171182560
Chr1	83.619	Marker26184	172143887
Chr1	83.619	Marker26186	172160879
Chr1	85.663	Marker26279	172525137
Chr1	85.663	Marker26552	174813221
Chr1	85.663	Marker26626	175655976
Chr1	86.064	Marker26734	176959678
Chr1	86.87	Marker26806	177436965
Chr1	86.87	Marker26898	178221343
Chr1	86.982	Marker26745	177013344
Chr1	86.982	Marker26961	178665273
Chr1	86.982	Marker27108	179423410
Chr1	87.529	Marker27325	180258524
Chr1	87.676	Marker26959	178664969
Chr1	88.077	Marker26899	178225306
Chr1	88.077	Marker27097	179324789
Chr1	88.077	Marker27412	180686722
Chr1	88.077	Marker27473	181375493
Chr1	88.486	Marker27100	179325016
Chr1	88.894	Marker27465	181346459
Chr1	89.303	Marker27516	181894507
Chr1	90.125	Marker27499	181439081
Chr1	90.125	Marker27521	181899395
Chr1	90.946	Marker27533	181982423
Chr1	91.355	Marker27606	182184714
Chr1	91.355	Marker27608	182189501
Chr1	91.355	Marker27792	183209824
Chr1	91.355	Marker27804	183671729
Chr1	91.82	Marker27597	182120690
Chr1	91.82	Marker27694	182725887
Chr1	91.82	Marker27697	182726085
Chr1	92.052	Marker27903	184064937
Chr1	92.748	Marker27904	184065011
Chr1	92.748	Marker28124	185429496
Chr1	93.096	Marker28251	185836050
Chr1	93.443	Marker27953	184320981
Chr1	93.443	Marker28564	188039998
Chr1	93.908	Marker28363	186511731
Chr1	93.908	Marker28381	186550883
Chr1	93.908	Marker28401	186670784

Chr1	93.908	Marker28637	188337311
Chr1	97.293	Marker28591	188103811
Chr1	97.293	Marker28798	190647657
Chr1	98.127	Marker28803	190686974
Chr1	99.804	Marker28930	191485904
Chr1	99.804	Marker28933	191486129
Chr1	99.804	Marker29012	191875952
Chr1	99.804	Marker29016	191876024
Chr1	99.804	Marker29295	194033699
Chr1	99.804	Marker29337	194211969
Chr1	99.804	Marker29340	194212206
Chr1	99.804	Marker29432	194909412
Chr1	99.804	Marker29772	197640751
Chr1	99.804	Marker30224	200965664
Chr1	99.804	Marker30437	202950908
Chr1	100.578	Marker28805	190687033
Chr1	100.578	Marker28911	191422862
Chr1	100.578	Marker28914	191423120
Chr1	105.494	Marker28951	191527199
Chr1	105.494	Marker29019	191905758
Chr1	105.824	Marker29737	197516697
Chr1	105.824	Marker29753	197581653
Chr1	105.824	Marker29988	199080330
Chr1	105.824	Marker29991	199105258
Chr1	105.824	Marker30129	200471971
Chr1	105.824	Marker30150	200551000
Chr1	105.824	Marker30581	204829006
Chr1	106.154	Marker30130	200472002
Chr1	106.154	Marker30232	201003823
Chr1	106.154	Marker30234	201005661
Chr1	106.154	Marker30236	201006134
Chr1	106.154	Marker30251	201039994
Chr1	106.154	Marker30252	201040034
Chr1	106.154	Marker30431	202937028
Chr1	106.154	Marker30439	202951087
Chr1	107.275	Marker30706	207325443
Chr1	107.275	Marker30885	209181957
Chr1	107.396	Marker30829	208404550
Chr1	107.645	Marker30886	209182190
Chr1	107.645	Marker31078	211058015
Chr1	107.645	Marker31114	211411089
Chr1	108.015	Marker30519	204174106
Chr1	108.015	Marker30656	207008102

Chr1	108.015	Marker30661	207069696
Chr1	112.612	Marker31407	215114171
Chr1	112.612	Marker31944	220674879
Chr1	114.39	Marker31118	211432280
Chr1	114.39	Marker31122	211443405
Chr1	115.443	Marker31642	218111129
Chr1	115.443	Marker31715	218852899
Chr1	115.999	Marker31707	218736802
Chr1	115.999	Marker31716	218853107
Chr1	115.999	Marker31977	220962393
Chr1	115.999	Marker32031	221617142
Chr1	116.7	Marker31498	216140510
Chr1	116.7	Marker31990	221048807
Chr1	116.7	Marker32141	222830191
Chr1	117.63	Marker32060	221979372
Chr1	117.63	Marker32080	222230141
Chr2	0	Marker33171	9024596
Chr2	0	Marker33829	13283044
Chr2	0	Marker34081	15147220
Chr2	0	Marker34083	15192512
Chr2	0	Marker34093	15236902
Chr2	0	Marker34096	15237147
Chr2	0	Marker34271	16675171
Chr2	0.857	Marker32220	844759
Chr2	0.857	Marker32335	1488520
Chr2	0.93	Marker34393	17308613
Chr2	1.32	Marker32197	822593
Chr2	1.32	Marker32359	1506292
Chr2	1.32	Marker32440	2003673
Chr2	1.32	Marker32509	2998732
Chr2	2.808	Marker32545	3597507
Chr2	2.808	Marker32598	4179763
Chr2	2.808	Marker32608	4180020
Chr2	3.271	Marker32689	4639584
Chr2	3.734	Marker32745	5430696
Chr2	3.734	Marker32778	5621888
Chr2	3.734	Marker32822	6279959
Chr2	3.734	Marker34420	17466425
Chr2	5.15	Marker32675	4508355
Chr2	5.15	Marker32716	5208716
Chr2	5.613	Marker32847	6414705
Chr2	5.613	Marker33516	11661593
Chr2	5.613	Marker34542	18980920

Chr2	5.613	Marker34546	18981147
Chr2	5.613	Marker34620	19862618
Chr2	7.681	Marker35543	25188701
Chr2	7.681	Marker35586	25735730
Chr2	9.75	Marker35824	27942790
Chr2	9.75	Marker35828	27943060
Chr2	10.649	Marker34282	16693514
Chr2	10.649	Marker34291	16717769
Chr2	11.731	Marker34564	19094889
Chr2	11.731	Marker34636	20318025
Chr2	11.731	Marker34837	21523102
Chr2	11.731	Marker34897	21864467
Chr2	11.731	Marker34905	22055701
Chr2	12.674	Marker35083	23148484
Chr2	12.674	Marker35089	23194211
Chr2	12.674	Marker35112	23285796
Chr2	12.674	Marker35154	23412797
Chr2	12.674	Marker35155	23413022
Chr2	12.674	Marker35158	23434195
Chr2	12.674	Marker35293	23844166
Chr2	13.362	Marker35285	23822390
Chr2	13.362	Marker35375	24156318
Chr2	13.362	Marker35391	24230520
Chr2	13.362	Marker35416	24570271
Chr2	13.901	Marker36117	29340292
Chr2	13.901	Marker36214	30200238
Chr2	14.742	Marker36819	34018310
Chr2	14.742	Marker36994	35249750
Chr2	14.983	Marker36255	30631428
Chr2	14.983	Marker36285	30827928
Chr2	14.983	Marker36590	33282266
Chr2	15.558	Marker36404	31922298
Chr2	15.558	Marker36886	34474600
Chr2	15.558	Marker36946	34889860
Chr2	15.558	Marker36950	34976933
Chr2	15.558	Marker37599	39375534
Chr2	17.205	Marker37902	40983081
Chr2	17.205	Marker38017	41584604
Chr2	17.205	Marker38096	41856034
Chr2	17.205	Marker38102	41858852
Chr2	17.205	Marker38173	42378292
Chr2	17.205	Marker38493	44096786
Chr2	17.205	Marker38500	44133076

Chr2	17.205	Marker38567	44714393
Chr2	17.205	Marker38676	45692567
Chr2	17.611	Marker37922	41097026
Chr2	17.611	Marker38010	41583726
Chr2	17.727	Marker36387	31647935
Chr2	17.727	Marker36539	33012458
Chr2	17.727	Marker36902	34586080
Chr2	17.727	Marker36957	35015592
Chr2	17.727	Marker37024	35462751
Chr2	17.727	Marker37025	35465699
Chr2	18.017	Marker37038	35489336
Chr2	18.017	Marker37106	36100939
Chr2	18.017	Marker37107	36101092
Chr2	19.912	Marker37182	36866940
Chr2	19.912	Marker37256	37428490
Chr2	20.994	Marker37625	39532878
Chr2	20.994	Marker37774	40447463
Chr2	20.994	Marker37861	40751894
Chr2	20.994	Marker37865	40757943
Chr2	20.994	Marker37869	40758252
Chr2	20.994	Marker37927	41169391
Chr2	24.303	Marker38131	42066748
Chr2	24.303	Marker38202	42600442
Chr2	24.303	Marker38405	43652744
Chr2	24.842	Marker38714	46118219
Chr2	25.38	Marker38641	45320022
Chr2	25.38	Marker38952	47278387
Chr2	25.38	Marker39085	49037489
Chr2	25.919	Marker39491	51192335
Chr2	26.457	Marker39820	54529363
Chr2	26.457	Marker39848	54668501
Chr2	26.923	Marker39921	55129481
Chr2	26.923	Marker39937	55143674
Chr2	27.388	Marker40123	56269440
Chr2	27.388	Marker40168	56593576
Chr2	28.327	Marker40263	58245460
Chr2	28.327	Marker40672	61433765
Chr2	28.327	Marker40711	61529096
Chr2	28.327	Marker40765	61656297
Chr2	28.327	Marker40838	62778828
Chr2	28.327	Marker40924	63495117
Chr2	28.327	Marker40931	63522118
Chr2	28.327	Marker40945	63656277

Chr2	28.327	Marker40970	63785147
Chr2	28.327	Marker40979	63851524
Chr2	28.327	Marker41515	66871821
Chr2	29.64	Marker41501	66794580
Chr2	29.64	Marker41516	66872156
Chr2	30.511	Marker41639	68383407
Chr2	30.511	Marker41786	69222674
Chr2	30.511	Marker41796	69250660
Chr2	30.944	Marker41907	69924882
Chr2	31.378	Marker41286	65838162
Chr2	31.378	Marker41909	69925151
Chr2	31.378	Marker42039	70512981
Chr2	31.378	Marker42042	70513249
Chr2	33.045	Marker42442	73711455
Chr2	33.045	Marker42547	74329093
Chr2	33.045	Marker42648	74696501
Chr2	33.045	Marker42850	75654080
Chr2	33.045	Marker42913	75928924
Chr2	33.045	Marker42921	75929220
Chr2	33.045	Marker43054	76199572
Chr2	33.045	Marker43096	76294949
Chr2	33.045	Marker43295	77999942
Chr2	34.547	Marker43097	76295154
Chr2	34.547	Marker43125	76594566
Chr2	34.547	Marker44271	85256973
Chr2	38.056	Marker43917	83111766
Chr2	38.056	Marker43971	83641927
Chr2	38.056	Marker43976	83642187
Chr2	38.056	Marker43994	83673077
Chr2	41.646	Marker44277	85298265
Chr2	42.518	Marker44065	83805570
Chr2	42.518	Marker44363	86758231
Chr2	42.518	Marker44470	87094218
Chr2	42.518	Marker44580	87614406
Chr2	42.518	Marker44753	89764289
Chr2	42.518	Marker44811	90113234
Chr2	42.518	Marker44840	90126572
Chr2	43.225	Marker45210	92356508
Chr2	43.561	Marker45268	92642250
Chr2	43.561	Marker45575	94366972
Chr2	43.931	Marker45381	93490454
Chr2	43.931	Marker45502	94033084
Chr2	44.637	Marker45004	91188513

Chr2	45.344	Marker45581	94367008
Chr2	45.344	Marker45674	94934793
Chr2	45.344	Marker45884	95699293
Chr2	46.409	Marker46156	97253843
Chr2	46.409	Marker46474	99046007
Chr2	46.763	Marker46159	97259213
Chr2	46.763	Marker46199	97386702
Chr2	48.902	Marker47043	101678060
Chr2	48.902	Marker47284	102430423
Chr2	48.902	Marker47391	102746532
Chr2	48.902	Marker47417	102771668
Chr2	48.902	Marker47509	103005184
Chr2	49.629	Marker48575	109119581
Chr2	49.629	Marker48683	109866256
Chr2	50.321	Marker47713	104258477
Chr2	50.321	Marker47734	104361137
Chr2	50.454	Marker47733	104360963
Chr2	50.454	Marker47825	104799643
Chr2	51.194	Marker48404	108067519
Chr2	51.935	Marker48135	106989600
Chr2	51.935	Marker48539	108595395
Chr2	52.675	Marker47999	105922569
Chr2	52.675	Marker48027	106258991
Chr2	52.675	Marker48028	106259011
Chr2	52.675	Marker48111	106976833
Chr2	52.675	Marker48275	107467481
Chr2	53.815	Marker48872	111490441
Chr2	53.815	Marker49036	112688447
Chr2	55.909	Marker49276	115202860
Chr2	55.909	Marker49303	115331397
Chr2	57.901	Marker49412	115654685
Chr2	57.901	Marker49578	117816485
Chr2	57.901	Marker49582	117826322
Chr2	57.901	Marker49644	118197389
Chr2	57.901	Marker49667	118369935
Chr2	57.901	Marker49688	118508401
Chr2	57.901	Marker49724	118582157
Chr2	57.901	Marker49937	120355359
Chr2	60.157	Marker49951	120470168
Chr2	60.157	Marker50130	121515545
Chr2	60.734	Marker50071	121035952
Chr2	60.734	Marker50075	121036208
Chr2	61.667	Marker50658	125506882

Chr2	61.667	Marker50854	126505315
Chr2	61.667	Marker50867	126527095
Chr2	61.667	Marker50905	126758652
Chr2	61.667	Marker51001	127181904
Chr2	61.667	Marker51023	127538461
Chr2	62.363	Marker50222	122776974
Chr2	62.363	Marker50332	124169892
Chr2	62.363	Marker50348	124442673
Chr2	62.363	Marker50817	126267986
Chr2	62.363	Marker50818	126268030
Chr2	65.159	Marker50823	126404556
Chr2	65.159	Marker51389	130161052
Chr2	66.073	Marker51038	127667877
Chr2	66.073	Marker51151	128368437
Chr2	66.528	Marker51578	131497390
Chr2	66.528	Marker51950	133094660
Chr2	66.528	Marker52011	133744308
Chr2	66.528	Marker52267	135277617
Chr2	66.528	Marker52601	138674638
Chr2	66.983	Marker51089	128082873
Chr2	66.983	Marker51334	129336706
Chr2	67.438	Marker51956	133190031
Chr2	67.438	Marker52014	133791345
Chr2	67.438	Marker52018	133815186
Chr2	67.438	Marker52111	134327608
Chr2	67.438	Marker52252	135110893
Chr2	67.438	Marker52489	138024561
Chr2	67.438	Marker52495	138032161
Chr2	67.438	Marker52580	138642501
Chr2	68.328	Marker51329	129301404
Chr2	68.328	Marker51468	130872611
Chr2	68.328	Marker51870	132636173
Chr2	68.328	Marker51873	132651547
Chr2	68.799	Marker52783	139443942
Chr2	68.799	Marker52847	141018897
Chr2	69.27	Marker53219	142609935
Chr2	69.27	Marker53355	142776875
Chr2	70.217	Marker53809	144938026
Chr2	70.233	Marker52886	141070642
Chr2	70.233	Marker53133	142221058
Chr2	70.233	Marker53360	142777108
Chr2	70.233	Marker53370	142810480
Chr2	70.688	Marker53002	141471603

Chr2	70.688	Marker53788	144823449
Chr2	70.688	Marker53817	145063154
Chr2	70.688	Marker53838	145259313
Chr2	71.385	Marker53484	143102046
Chr2	71.385	Marker53533	143470278
Chr2	71.385	Marker53636	143780347
Chr2	72.082	Marker53638	143782168
Chr2	72.082	Marker54044	145891660
Chr2	72.082	Marker54543	148586527
Chr2	72.082	Marker54794	149321914
Chr2	72.547	Marker54135	146134465
Chr2	73.477	Marker54203	146775259
Chr2	73.477	Marker54295	147394427
Chr2	73.851	Marker53895	145308643
Chr2	73.851	Marker54778	149268144
Chr2	73.851	Marker54798	149322140
Chr2	73.851	Marker54816	149362075
Chr2	73.851	Marker55001	150030700
Chr2	73.851	Marker55013	150039995
Chr2	73.851	Marker55015	150040209
Chr2	73.851	Marker55087	150478742
Chr2	74.225	Marker55096	150526906
Chr2	74.225	Marker55101	150527136
Chr2	74.225	Marker55103	150533833
Chr2	74.225	Marker55140	150626611
Chr2	74.225	Marker55264	150952416
Chr2	76.919	Marker55711	153350386
Chr2	76.919	Marker55787	153866849
Chr2	77.208	Marker55293	151534862
Chr2	77.208	Marker55352	151842431
Chr2	77.208	Marker55377	151914671
Chr2	77.208	Marker55511	152461064
Chr2	77.671	Marker55644	153082166
Chr2	77.815	Marker55701	153349054
Chr2	77.815	Marker55784	153808016
Chr2	78.423	Marker55274	151257898
Chr2	78.423	Marker55276	151258116
Chr2	78.423	Marker55300	151627258
Chr2	78.423	Marker55506	152460862
Chr2	78.423	Marker55556	152787567
Chr2	78.423	Marker55571	152855840
Chr2	78.423	Marker55594	152924659
Chr2	78.423	Marker55618	153013432

Chr2	78.423	Marker55634	153061379
Chr2	78.423	Marker55718	153387925
Chr2	78.423	Marker55947	154705827
Chr2	78.423	Marker55952	154707630
Chr2	78.423	Marker56012	155022808
Chr2	78.654	Marker55267	150953040
Chr2	78.654	Marker55572	152856153
Chr2	80.548	Marker56220	158018883
Chr2	80.548	Marker56222	158025067
Chr2	80.548	Marker56227	158041015
Chr2	81.192	Marker56367	159325023
Chr2	81.192	Marker56784	161630924
Chr2	81.192	Marker56785	161631166
Chr2	81.192	Marker56914	162477564
Chr2	83.402	Marker56926	162639922
Chr2	83.75	Marker56956	162658063
Chr2	83.75	Marker57144	163571602
Chr2	84.095	Marker56932	162640196
Chr2	84.098	Marker57196	163743739
Chr2	84.098	Marker57262	164044779
Chr2	84.098	Marker57326	164435581
Chr2	84.445	Marker57193	163739961
Chr2	85.855	Marker57181	163734954
Chr2	85.855	Marker57190	163735246
Chr2	87.626	Marker57748	168385840
Chr2	87.626	Marker57797	168875677
Chr2	87.626	Marker57809	168901012
Chr2	87.626	Marker57812	168903452
Chr2	87.626	Marker57829	168989197
Chr2	87.626	Marker58357	173795551
Chr2	88.127	Marker57151	163592301
Chr2	88.627	Marker57344	164528130
Chr2	88.627	Marker57517	165916178
Chr2	88.627	Marker57884	169357673
Chr2	88.627	Marker57935	169572650
Chr2	90.656	Marker58214	172506517
Chr2	91.156	Marker58350	173543643
Chr2	91.656	Marker57999	170764546
Chr2	91.656	Marker58231	172760912
Chr2	91.656	Marker58352	173563966
Chr2	92.108	Marker58447	175173226
Chr2	92.559	Marker58597	175954528
Chr2	94.076	Marker59276	182927313

Chr2	94.076	Marker59998	190299889
Chr2	94.076	Marker60049	190561038
Chr2	94.076	Marker60050	190578525
Chr2	94.076	Marker60241	192995606
Chr2	98.276	Marker60048	190560858
Chr2	98.728	Marker59961	190086558
Chr2	99.18	Marker59808	189191495
Chr2	100.087	Marker60052	190633380
Chr2	100.087	Marker60120	191362349
Chr2	100.087	Marker60160	191781709
Chr2	100.611	Marker60760	196994130
Chr2	100.611	Marker60791	197057556
Chr2	101.662	Marker60574	195470871
Chr2	101.771	Marker60404	194674203
Chr2	101.771	Marker61113	199117575
Chr2	102.186	Marker60353	194105489
Chr2	102.186	Marker60411	194803783
Chr2	102.186	Marker60426	194902386
Chr2	102.649	Marker60484	195268910
Chr2	102.649	Marker60578	195473213
Chr2	103.116	Marker60699	196488640
Chr2	103.116	Marker61133	199137178
Chr2	106.478	Marker61397	200446433
Chr2	106.478	Marker61502	201938812
Chr2	106.478	Marker61548	202453313
Chr2	107.446	Marker61686	204353656
Chr2	107.446	Marker61852	205962989
Chr2	109.323	Marker62283	210630560
Chr2	109.323	Marker62372	211463290
Chr3	0	Marker154172	1706938
Chr3	0	Marker154173	1707233
Chr3	0	Marker154316	3239968
Chr3	1.197	Marker154319	3288399
Chr3	1.604	Marker154317	3240038
Chr3	1.604	Marker154345	3628278
Chr3	1.74	Marker154358	3769866
Chr3	3.305	Marker154730	7126501
Chr3	3.305	Marker154736	7217069
Chr3	4.145	Marker154357	3733284
Chr3	4.145	Marker154359	3770110
Chr3	4.702	Marker154514	4923258
Chr3	4.702	Marker154735	7133185
Chr3	4.702	Marker154784	7916695

Chr3	4.702	Marker154788	7917938
Chr3	4.702	Marker154791	7918205
Chr3	4.702	Marker154795	7922208
Chr3	4.702	Marker154940	8515958
Chr3	4.702	Marker154941	8516006
Chr3	4.702	Marker155409	12106818
Chr3	5.167	Marker154886	8289629
Chr3	5.633	Marker154792	7921995
Chr3	5.633	Marker154973	8705712
Chr3	5.95	Marker155043	9153807
Chr3	5.95	Marker155053	9187646
Chr3	5.95	Marker155175	9885918
Chr3	6.268	Marker155264	10829282
Chr3	7.231	Marker155535	13021515
Chr3	7.231	Marker155648	14669883
Chr3	7.231	Marker155649	14670144
Chr3	7.231	Marker155696	15180176
Chr3	7.549	Marker155909	16707290
Chr3	7.867	Marker156258	20819861
Chr3	8.185	Marker155688	15113086
Chr3	8.185	Marker155842	16177574
Chr3	8.185	Marker155892	16678069
Chr3	8.185	Marker155917	16728842
Chr3	8.185	Marker155918	16734473
Chr3	8.185	Marker156131	19401269
Chr3	9.088	Marker155414	12107121
Chr3	9.088	Marker155441	12322391
Chr3	16.133	Marker156257	20813590
Chr3	16.133	Marker156278	20989818
Chr3	16.133	Marker156406	23174067
Chr3	16.133	Marker156427	23316839
Chr3	16.133	Marker156434	23350522
Chr3	16.133	Marker156467	24011504
Chr3	16.133	Marker156547	24776775
Chr3	16.133	Marker156897	29459514
Chr3	16.985	Marker157442	35419071
Chr3	16.985	Marker157877	41060836
Chr3	16.985	Marker157892	41256770
Chr3	22.025	Marker157194	32317297
Chr3	22.025	Marker157335	34756633
Chr3	23.645	Marker157907	41431504
Chr3	25.265	Marker157936	41760863
Chr3	25.265	Marker158047	42325818

Chr3	28.632	Marker158151	43050040
Chr3	28.632	Marker158171	43079839
Chr3	30.3	Marker158321	44887396
Chr3	31.044	Marker158386	45570509
Chr3	32.813	Marker158891	51124380
Chr3	32.813	Marker158908	51401679
Chr3	32.813	Marker158910	51411857
Chr3	32.813	Marker159165	53055451
Chr3	32.813	Marker159305	54041041
Chr3	32.813	Marker159348	54278782
Chr3	32.813	Marker159591	56900729
Chr3	33.643	Marker159381	54466931
Chr3	37.011	Marker159596	56901054
Chr3	37.011	Marker159757	57323265
Chr3	37.011	Marker160013	59580572
Chr3	38.678	Marker160202	61077938
Chr3	41.192	Marker160317	61695490
Chr3	41.192	Marker160398	62092960
Chr3	42.022	Marker160742	65603441
Chr3	42.852	Marker161229	70955543
Chr3	42.852	Marker161393	72020851
Chr3	46.219	Marker161545	72850590
Chr3	46.219	Marker161582	72895444
Chr3	48.733	Marker161754	73616925
Chr3	49.563	Marker161885	74120769
Chr3	50.393	Marker162275	77875177
Chr3	50.393	Marker162327	78536879
Chr3	51.223	Marker162356	78759797
Chr3	51.294	Marker160396	62082512
Chr3	52.053	Marker162563	80942706
Chr3	52.053	Marker162597	81735980
Chr3	52.053	Marker162600	81736034
Chr3	52.053	Marker162626	81993389
Chr3	52.053	Marker162631	82035962
Chr3	52.053	Marker162636	82036197
Chr3	52.053	Marker162686	82476262
Chr3	52.93	Marker160522	63331073
Chr3	52.93	Marker161447	72291692
Chr3	53.575	Marker161493	72463393
Chr3	53.575	Marker161625	72967473
Chr3	53.575	Marker161686	73366881
Chr3	53.896	Marker161695	73373362
Chr3	54.217	Marker162117	76224797

Chr3	54.538	Marker161935	74951303
Chr3	54.859	Marker162460	80072232
Chr3	54.859	Marker162571	81159730
Chr3	56.831	Marker162825	84196370
Chr3	57.152	Marker162771	83953605
Chr3	57.152	Marker163299	88110709
Chr3	57.152	Marker163554	90755724
Chr3	57.152	Marker163558	90756907
Chr3	57.152	Marker163586	90948277
Chr3	57.152	Marker164142	94180511
Chr3	57.152	Marker164158	94381657
Chr3	57.152	Marker164202	94863601
Chr3	57.152	Marker164500	96007548
Chr3	57.152	Marker164577	96299506
Chr3	57.152	Marker164704	97294631
Chr3	57.85	Marker165356	99734918
Chr3	57.85	Marker165539	100862391
Chr3	57.85	Marker165540	100863496
Chr3	58.198	Marker164584	96714419
Chr3	58.545	Marker164790	97532385
Chr3	58.545	Marker164835	97580019
Chr3	58.545	Marker165350	99717566
Chr3	59.011	Marker165489	100313477
Chr3	59.011	Marker165939	104391516
Chr3	59.011	Marker165976	104439495
Chr3	59.011	Marker166248	105667873
Chr3	59.948	Marker165643	101645416
Chr3	59.948	Marker165798	103241552
Chr3	59.948	Marker165850	103721317
Chr3	59.948	Marker165888	104111879
Chr3	60.414	Marker166232	105657859
Chr3	60.88	Marker166301	106081977
Chr3	61.346	Marker166302	106082024
Chr3	61.346	Marker166467	106821283
Chr3	61.346	Marker166690	107934755
Chr3	61.346	Marker166742	108292655
Chr3	61.346	Marker167377	116981940
Chr3	61.346	Marker167429	117526018
Chr3	61.346	Marker167580	119249192
Chr3	61.346	Marker168037	122269505
Chr3	61.346	Marker168144	123603337
Chr3	61.835	Marker166818	110289372
Chr3	61.835	Marker166902	111151097

Chr3	62.758	Marker167315	116456375
Chr3	62.758	Marker168236	124515042
Chr3	62.758	Marker168239	124524078
Chr3	64.119	Marker167938	121364290
Chr3	64.768	Marker168856	131042452
Chr3	66.853	Marker168506	128444865
Chr3	66.853	Marker168577	129340433
Chr3	66.853	Marker168660	130406597
Chr3	66.853	Marker169024	131880606
Chr3	68.213	Marker168859	131045120
Chr3	68.213	Marker169168	132631305
Chr3	69.574	Marker169169	132655971
Chr3	69.574	Marker169170	132656003
Chr3	69.574	Marker169207	133243045
Chr3	70.395	Marker169145	132475690
Chr3	70.395	Marker169348	133991473
Chr3	70.395	Marker169478	135357454
Chr3	70.395	Marker169578	136486760
Chr3	71.216	Marker169615	137681057
Chr3	71.216	Marker169748	138124694
Chr3	71.216	Marker169963	139300394
Chr3	74.204	Marker170008	139745586
Chr3	74.204	Marker170021	139867336
Chr3	74.204	Marker170061	140198183
Chr3	74.618	Marker170097	140542209
Chr3	74.618	Marker170391	142305601
Chr3	74.618	Marker170392	142347352
Chr3	77.847	Marker170192	141098669
Chr3	77.847	Marker170225	141208343
Chr3	79.972	Marker170279	141522626
Chr3	79.972	Marker170289	141565548
Chr3	79.972	Marker170368	142210563
Chr3	82.098	Marker170430	142779834
Chr3	83.15	Marker170450	143142190
Chr3	83.15	Marker170453	143161761
Chr3	83.674	Marker170529	144095186
Chr3	83.674	Marker171339	149391484
Chr3	83.745	Marker171361	149597928
Chr3	83.745	Marker172210	157179471
Chr3	84.198	Marker171732	153152069
Chr3	84.722	Marker171838	154400245
Chr3	85.246	Marker171806	154006309
Chr3	85.769	Marker172393	158508499

Chr3	85.769	Marker172553	159728520
Chr3	86.293	Marker172574	159913550
Chr3	86.293	Marker172661	160437430
Chr3	86.293	Marker172914	161979058
Chr3	86.654	Marker173030	163013657
Chr3	87.014	Marker173366	166470793
Chr3	87.739	Marker173455	167212485
Chr3	87.739	Marker173530	167742134
Chr3	88.099	Marker173531	167777588
Chr3	89.191	Marker174084	174518422
Chr3	90.951	Marker172730	161417430
Chr3	90.951	Marker172961	162266109
Chr3	92.173	Marker175143	181142941
Chr3	92.173	Marker175335	182204915
Chr3	92.173	Marker175338	182205229
Chr3	92.173	Marker175641	183824255
Chr3	92.173	Marker175877	185919229
Chr3	92.173	Marker175882	185966077
Chr3	92.173	Marker175995	186587379
Chr3	92.173	Marker176023	186658099
Chr3	92.173	Marker176036	186671032
Chr3	92.173	Marker176066	186805430
Chr3	92.173	Marker176094	187223531
Chr3	92.173	Marker176105	187342480
Chr3	92.251	Marker173155	163934887
Chr3	92.898	Marker173533	167786479
Chr3	92.898	Marker173640	170548738
Chr3	92.898	Marker173727	171631940
Chr3	92.898	Marker174279	176409524
Chr3	94.21	Marker173850	172872879
Chr3	94.21	Marker174398	177212259
Chr3	94.533	Marker174519	177916927
Chr3	94.533	Marker174525	177917166
Chr3	94.533	Marker174558	178002031
Chr3	94.533	Marker174584	178145444
Chr3	94.533	Marker174848	179937620
Chr3	94.533	Marker174855	180016872
Chr3	94.533	Marker174859	180017134
Chr3	94.533	Marker174871	180041739
Chr3	94.533	Marker174887	180104128
Chr3	94.533	Marker175242	181834875
Chr3	94.996	Marker175296	182040251
Chr3	95.459	Marker175711	184189566

Chr3	95.459	Marker176009	186603338
Chr4	0	Marker176549	2506289
Chr4	0	Marker176755	3423892
Chr4	0	Marker176834	3863549
Chr4	0.93	Marker176144	409131
Chr4	0.93	Marker176178	747977
Chr4	0.93	Marker176388	1722922
Chr4	0.93	Marker176483	2193771
Chr4	1.402	Marker176169	696964
Chr4	1.402	Marker176376	1708488
Chr4	1.402	Marker176449	1913753
Chr4	1.402	Marker176831	3851546
Chr4	1.861	Marker177016	4546508
Chr4	1.861	Marker177066	4595467
Chr4	1.865	Marker177136	4898004
Chr4	2.328	Marker177249	6104681
Chr4	2.328	Marker177640	8684209
Chr4	2.328	Marker177661	8851864
Chr4	2.328	Marker177899	10170438
Chr4	2.791	Marker176942	4222741
Chr4	2.791	Marker178261	12741400
Chr4	2.791	Marker178428	13956794
Chr4	3.254	Marker177133	4897723
Chr4	3.254	Marker177257	6114789
Chr4	3.254	Marker177264	6127296
Chr4	3.254	Marker177267	6134470
Chr4	3.254	Marker177590	8474631
Chr4	3.254	Marker177705	9242504
Chr4	3.254	Marker177761	9459407
Chr4	3.254	Marker177874	10120978
Chr4	3.254	Marker177876	10121034
Chr4	3.527	Marker177094	4654272
Chr4	3.527	Marker177223	6027508
Chr4	4.776	Marker178079	11474164
Chr4	4.776	Marker178505	14193087
Chr4	4.776	Marker178516	14234687
Chr4	4.776	Marker178721	15726321
Chr4	4.918	Marker177990	10953169
Chr4	6.025	Marker178398	13423285
Chr4	6.298	Marker178431	14048181
Chr4	6.298	Marker178967	16905696
Chr4	6.298	Marker179287	18749495
Chr4	6.763	Marker178519	14235888

Chr4	6.763	Marker178521	14236139
Chr4	6.763	Marker178584	14825633
Chr4	7.478	Marker179435	19870255
Chr4	7.478	Marker179706	21009703
Chr4	8.915	Marker179867	21548961
Chr4	10.351	Marker179892	21831476
Chr4	10.351	Marker180247	24568267
Chr4	11.787	Marker180090	22902432
Chr4	16.007	Marker179683	20828955
Chr4	16.007	Marker180334	25119417
Chr4	18.472	Marker180404	25459688
Chr4	18.472	Marker180460	26021441
Chr4	20.35	Marker180482	26260221
Chr4	20.35	Marker180580	27196375
Chr4	20.35	Marker180724	28145358
Chr4	21.444	Marker180498	26525700
Chr4	21.986	Marker180715	28134546
Chr4	21.986	Marker180859	29860491
Chr4	22.218	Marker180835	29457839
Chr4	23.622	Marker180906	30828816
Chr4	23.622	Marker181072	33520470
Chr4	23.622	Marker181194	34877793
Chr4	23.853	Marker181096	33628520
Chr4	24.085	Marker181146	34698742
Chr4	24.085	Marker181359	36425887
Chr4	25.024	Marker181124	34340442
Chr4	25.024	Marker181361	36435161
Chr4	25.024	Marker181460	36776390
Chr4	25.024	Marker181477	36953566
Chr4	25.963	Marker181143	34686293
Chr4	25.963	Marker181560	37851562
Chr4	25.963	Marker181604	38560534
Chr4	27.85	Marker181627	38624807
Chr4	28.315	Marker181631	38641629
Chr4	28.315	Marker181684	39161224
Chr4	29.983	Marker181639	38643606
Chr4	31.409	Marker181640	38816151
Chr4	33.027	Marker181674	39160926
Chr4	33.492	Marker181717	39383311
Chr4	33.492	Marker181821	39731381
Chr4	33.492	Marker181874	40071874
Chr4	33.492	Marker181953	40419387
Chr4	33.723	Marker181878	40072177

Chr4	34.128	Marker181914	40273573
Chr4	34.128	Marker182267	43765339
Chr4	34.812	Marker182145	42047740
Chr4	36.063	Marker182276	43941590
Chr4	36.615	Marker182523	46228391
Chr4	36.615	Marker182539	46248880
Chr4	36.615	Marker182540	46249168
Chr4	36.615	Marker182632	47105373
Chr4	36.937	Marker183627	60613948
Chr4	37.126	Marker182310	44268941
Chr4	37.126	Marker182361	44595339
Chr4	37.126	Marker182370	44630064
Chr4	37.914	Marker183629	60614223
Chr4	37.914	Marker184038	63716857
Chr4	39.61	Marker182717	48068872
Chr4	40.43	Marker183626	60588129
Chr4	41.25	Marker183849	62539661
Chr4	42.07	Marker183884	62739634
Chr4	42.89	Marker183899	62764696
Chr4	42.89	Marker184161	64715379
Chr4	46.216	Marker184303	65872802
Chr4	46.216	Marker184841	69691429
Chr4	47.037	Marker184795	69317839
Chr4	47.037	Marker184796	69318045
Chr4	48.69	Marker184757	69232994
Chr4	48.69	Marker184759	69233045
Chr4	48.69	Marker185070	71442794
Chr4	49.391	Marker184306	65888848
Chr4	49.391	Marker184437	67009136
Chr4	49.391	Marker184531	67536495
Chr4	49.391	Marker184532	67542468
Chr4	49.391	Marker184539	67629387
Chr4	49.391	Marker184667	68262777
Chr4	49.391	Marker184711	68695454
Chr4	49.391	Marker184747	69192499
Chr4	49.391	Marker184835	69673213
Chr4	49.391	Marker184851	69742240
Chr4	49.391	Marker184958	70212418
Chr4	49.391	Marker185072	71443051
Chr4	49.391	Marker185202	72288590
Chr4	49.391	Marker185205	72342869
Chr4	49.856	Marker185278	72811449
Chr4	50.204	Marker185499	74179172

Chr4	50.551	Marker185725	76582729
Chr4	50.551	Marker185781	76885350
Chr4	50.551	Marker185789	76910192
Chr4	51.721	Marker185281	72812487
Chr4	51.721	Marker185297	72959527
Chr4	51.721	Marker185465	73876369
Chr4	51.721	Marker185922	77281072
Chr4	51.721	Marker186522	83436471
Chr4	51.721	Marker186625	84047380
Chr4	51.721	Marker186632	84079567
Chr4	51.721	Marker186720	85542841
Chr4	51.721	Marker186734	85721965
Chr4	51.721	Marker186737	85758998
Chr4	52.652	Marker185920	77280863
Chr4	52.652	Marker186199	80579832
Chr4	52.883	Marker186108	79305827
Chr4	52.883	Marker186279	81501079
Chr4	52.883	Marker186336	82669406
Chr4	52.883	Marker186483	83309103
Chr4	53.115	Marker186494	83358500
Chr4	53.816	Marker185809	76998751
Chr4	53.816	Marker186112	79344233
Chr4	53.816	Marker186515	83429855
Chr4	54.512	Marker186516	83433361
Chr4	55.092	Marker186739	85759168
Chr4	55.286	Marker186572	83642956
Chr4	55.286	Marker186710	85173282
Chr4	55.672	Marker186576	83643217
Chr4	55.672	Marker186823	86442890
Chr4	55.903	Marker186761	85920273
Chr4	55.903	Marker186762	85921329
Chr4	55.903	Marker186789	86190555
Chr4	56.884	Marker187186	90506336
Chr4	57.208	Marker187092	88827934
Chr4	57.208	Marker187194	90536732
Chr4	57.208	Marker187218	90589129
Chr4	57.208	Marker187240	90680741
Chr4	57.532	Marker187279	90837511
Chr4	57.532	Marker187293	91013237
Chr4	57.532	Marker187464	92996613
Chr4	57.532	Marker187571	93496124
Chr4	57.532	Marker187579	93511507
Chr4	57.532	Marker187601	93540512

Chr4	59.12	Marker187580	93524409
Chr4	59.12	Marker187633	93633981
Chr4	59.12	Marker187635	93634201
Chr4	59.12	Marker187742	93952591
Chr4	59.12	Marker187879	94733121
Chr4	59.644	Marker187731	93943779
Chr4	59.644	Marker187748	93989595
Chr4	59.644	Marker187897	94828852
Chr4	59.644	Marker187974	95354152
Chr4	59.644	Marker188101	95947174
Chr4	59.644	Marker188386	97407776
Chr4	60.494	Marker188196	96517297
Chr4	60.494	Marker188464	97710148
Chr4	60.494	Marker188486	97801798
Chr4	60.494	Marker189429	102025355
Chr4	60.608	Marker188283	96912608
Chr4	60.926	Marker188520	97997523
Chr4	61.565	Marker188566	98141708
Chr4	61.883	Marker188616	98375099
Chr4	61.883	Marker188960	100363457
Chr4	61.883	Marker189003	100536536
Chr4	61.883	Marker189168	101156271
Chr4	61.883	Marker189277	101565468
Chr4	61.883	Marker189444	102160785
Chr4	62.201	Marker189643	102906722
Chr4	64.423	Marker189496	102340432
Chr4	64.94	Marker191173	116058389
Chr4	65.528	Marker189927	105777963
Chr4	65.528	Marker189997	105883716
Chr4	65.528	Marker190027	106105947
Chr4	65.528	Marker190030	106106167
Chr4	65.528	Marker190031	106122610
Chr4	65.528	Marker190122	107092718
Chr4	65.528	Marker190271	108571809
Chr4	66.34	Marker189930	105778033
Chr4	66.617	Marker190275	108572096
Chr4	66.617	Marker190614	113112043
Chr4	66.617	Marker190658	113292957
Chr4	66.617	Marker190941	115060159
Chr4	67.159	Marker191521	118037339
Chr4	67.979	Marker191983	120017418
Chr4	68.248	Marker191529	118039608
Chr4	68.791	Marker191723	119120488

Chr4	68.791	Marker192223	121650858
Chr4	69.721	Marker191497	117748111
Chr4	69.721	Marker191543	118070982
Chr4	69.721	Marker191545	118071010
Chr4	69.721	Marker191707	119029612
Chr4	69.721	Marker191797	119312473
Chr4	69.721	Marker192095	120841980
Chr4	69.721	Marker192096	120842014
Chr4	69.721	Marker192177	121208384
Chr4	69.721	Marker192252	121995463
Chr4	69.721	Marker192286	122164745
Chr4	70.183	Marker192670	124414253
Chr4	70.183	Marker192727	124757479
Chr4	70.183	Marker192819	125234843
Chr4	70.183	Marker192820	125235090
Chr4	70.184	Marker192321	122521618
Chr4	70.184	Marker192355	122592498
Chr4	70.648	Marker192407	122749575
Chr4	70.648	Marker192645	124295616
Chr4	70.648	Marker192669	124413953
Chr4	71.112	Marker192691	124479251
Chr4	71.112	Marker193050	127406476
Chr4	71.112	Marker193332	129204418
Chr4	71.112	Marker193334	129206335
Chr4	71.813	Marker192842	125879342
Chr4	72.514	Marker192877	126308666
Chr4	72.514	Marker193269	128867529
Chr4	74.052	Marker193402	129344986
Chr4	74.052	Marker193678	130735261
Chr4	75.259	Marker193318	129116288
Chr4	75.259	Marker193398	129332403
Chr4	75.59	Marker193434	129596939
Chr4	75.59	Marker193440	129624839
Chr4	75.59	Marker193738	131249207
Chr4	75.59	Marker193757	131592748
Chr4	75.977	Marker194431	135594251
Chr4	75.977	Marker194548	136314645
Chr4	77.149	Marker194739	137322740
Chr4	77.927	Marker194396	135422485
Chr4	77.927	Marker194434	135613435
Chr4	77.927	Marker194750	137367624
Chr4	77.927	Marker194757	137404016
Chr4	80.344	Marker194846	137853262

Chr4	80.344	Marker194849	137866382
Chr4	80.807	Marker194762	137418671
Chr4	80.807	Marker194830	137766630
Chr4	80.807	Marker194842	137819111
Chr4	81.27	Marker195143	142200603
Chr4	81.27	Marker195228	143215335
Chr4	81.746	Marker195288	143503768
Chr4	82.209	Marker195287	143472692
Chr4	82.209	Marker195343	143705702
Chr4	82.209	Marker195557	145400070
Chr4	82.209	Marker195623	145831558
Chr4	82.209	Marker195646	145922958
Chr4	82.672	Marker195116	142000915
Chr4	82.672	Marker195266	143398474
Chr4	82.672	Marker195313	143644145
Chr4	83.019	Marker195472	144708554
Chr4	83.367	Marker195519	145211526
Chr4	83.896	Marker195705	146516098
Chr4	84.425	Marker195976	148504875
Chr4	84.425	Marker197000	156924445
Chr4	85.463	Marker195662	145936373
Chr4	85.463	Marker196077	148999976
Chr4	85.875	Marker196082	149000262
Chr4	85.875	Marker196399	151404043
Chr4	85.875	Marker196520	152096203
Chr4	85.875	Marker196558	152459472
Chr4	87.121	Marker196868	155740126
Chr4	87.121	Marker196927	156504488
Chr4	87.121	Marker196971	156823877
Chr4	87.121	Marker196972	156824166
Chr4	87.121	Marker196991	156864945
Chr4	87.121	Marker196995	156865001
Chr4	87.121	Marker197067	157257500
Chr4	87.121	Marker197113	157456474
Chr4	87.353	Marker197077	157274098
Chr4	88.373	Marker197293	158551486
Chr4	88.373	Marker197311	158784590
Chr4	89.078	Marker197126	157474124
Chr4	89.078	Marker197226	157840686
Chr4	89.932	Marker197533	161047729
Chr4	89.932	Marker197567	161141699
Chr4	89.932	Marker197570	161142079
Chr4	90.962	Marker197605	161207625

Chr4	90.962	Marker197619	161263621
Chr4	90.962	Marker197651	161448545
Chr4	92.099	Marker197766	162248037
Chr4	92.099	Marker197809	162612270
Chr4	92.099	Marker197899	163264506
Chr4	92.953	Marker197843	162813926
Chr4	92.953	Marker197978	165179410
Chr4	97.393	Marker197922	163507836
Chr4	97.393	Marker198073	166013365
Chr4	97.393	Marker198123	166411389
Chr4	97.393	Marker198222	167398790
Chr4	97.393	Marker198228	167399008
Chr4	97.393	Marker198266	167729861
Chr4	97.393	Marker198409	169168254
Chr4	98.094	Marker198053	165853383
Chr4	98.094	Marker198419	169182972
Chr4	98.094	Marker198421	169183226
Chr4	99.736	Marker198591	170324395
Chr4	100.179	Marker198521	169957428
Chr4	100.179	Marker198593	170396770
Chr4	100.584	Marker199077	175170509
Chr4	101.812	Marker198634	171037286
Chr4	101.812	Marker198644	171081469
Chr4	101.812	Marker199072	175154502
Chr4	102.277	Marker199177	175785871
Chr4	102.821	Marker199193	175998694
Chr4	102.821	Marker199206	176107479
Chr4	102.821	Marker199676	178491338
Chr4	103.913	Marker199686	178526767
Chr4	103.913	Marker199698	178567335
Chr4	103.913	Marker199885	179401487
Chr4	105.092	Marker199437	177354670
Chr4	105.092	Marker199826	179146484
Chr4	105.092	Marker199940	179776419
Chr4	105.092	Marker200144	180712855
Chr4	105.324	Marker200006	180445409
Chr4	105.324	Marker201029	189984758
Chr4	105.789	Marker200442	183748763
Chr4	105.789	Marker200448	183749052
Chr4	105.789	Marker200658	185682626
Chr4	106.02	Marker200665	185743517
Chr4	106.02	Marker200677	185960507
Chr4	106.02	Marker200995	189891789

Chr4	107.899	Marker201075	191002948
Chr4	107.899	Marker201124	191825661
Chr4	107.899	Marker201155	192361556
Chr4	107.899	Marker201202	192676351
Chr4	108.362	Marker201244	192988430
Chr4	108.362	Marker201246	192994869
Chr5	0	Marker201570	1632509
Chr5	0	Marker201635	2134548
Chr5	0	Marker201777	2544798
Chr5	0	Marker202024	3822275
Chr5	0	Marker202353	6514511
Chr5	1.179	Marker202415	7606642
Chr5	1.645	Marker202430	7682013
Chr5	1.645	Marker202712	9780545
Chr5	2.009	Marker202525	8053497
Chr5	2.009	Marker202723	9911560
Chr5	2.278	Marker202604	8812796
Chr5	2.278	Marker202780	10186999
Chr5	2.742	Marker202795	10375763
Chr5	2.912	Marker202783	10187268
Chr5	4.185	Marker203220	15289818
Chr5	4.6	Marker202839	11052017
Chr5	4.819	Marker202829	11051799
Chr5	6.092	Marker202963	12475278
Chr5	8.011	Marker203507	20069279
Chr5	8.011	Marker203520	20097567
Chr5	8.011	Marker203604	21362955
Chr5	8.011	Marker203660	21844623
Chr5	8.942	Marker203810	22665475
Chr5	8.942	Marker203935	23250505
Chr5	8.942	Marker204089	25256807
Chr5	8.942	Marker204170	25808089
Chr5	9.888	Marker203802	22618875
Chr5	10.345	Marker204425	27590527
Chr5	10.818	Marker204416	27413058
Chr5	11.748	Marker204451	27738623
Chr5	11.748	Marker204556	28442422
Chr5	11.748	Marker204607	28601765
Chr5	13.152	Marker204633	28797495
Chr5	13.152	Marker204651	28859878
Chr5	13.152	Marker204797	29774266
Chr5	13.383	Marker204686	29029940
Chr5	13.964	Marker205171	33313945

Chr5	14.546	Marker205214	33631494
Chr5	14.546	Marker205241	33816382
Chr5	14.548	Marker205246	33854403
Chr5	14.548	Marker205307	34306812
Chr5	15.713	Marker205352	34676631
Chr5	15.713	Marker205432	35481567
Chr5	15.713	Marker205910	39531501
Chr5	15.713	Marker206069	40181742
Chr5	15.713	Marker206145	40894617
Chr5	15.713	Marker206250	42353200
Chr5	17.136	Marker205535	36488537
Chr5	17.136	Marker205636	37374415
Chr5	17.136	Marker205804	38915522
Chr5	17.367	Marker205821	38989406
Chr5	17.598	Marker205812	38922517
Chr5	17.598	Marker205854	39294120
Chr5	17.598	Marker206012	39958854
Chr5	17.598	Marker206263	42415073
Chr5	17.598	Marker206272	42539114
Chr5	18.804	Marker206261	42414835
Chr5	18.804	Marker206788	46637693
Chr5	19.704	Marker206633	45047613
Chr5	19.704	Marker207106	49289702
Chr5	20.738	Marker207600	53020553
Chr5	22.818	Marker207918	55805855
Chr5	22.818	Marker208007	56380034
Chr5	22.818	Marker208305	58766733
Chr5	23.28	Marker208288	58609996
Chr5	23.28	Marker208462	59471595
Chr5	23.28	Marker208501	59576381
Chr5	23.28	Marker208502	59659980
Chr5	23.28	Marker208515	59690968
Chr5	23.28	Marker208629	60116706
Chr5	23.28	Marker208684	60393693
Chr5	23.743	Marker208708	60427282
Chr5	24.205	Marker208908	63159558
Chr5	24.697	Marker208006	56379877
Chr5	24.697	Marker208476	59545849
Chr5	25.606	Marker208975	64391581
Chr5	27.007	Marker209338	67217302
Chr5	27.058	Marker208503	59660278
Chr5	27.058	Marker208563	59832702
Chr5	27.989	Marker209158	65618389

Chr5	27.989	Marker209611	70219297
Chr5	28.92	Marker209310	67031647
Chr5	28.92	Marker209629	70277494
Chr5	28.92	Marker209641	70296565
Chr5	29.849	Marker209719	71642003
Chr5	29.849	Marker209749	71683486
Chr5	30.323	Marker210712	82546378
Chr5	30.787	Marker210965	87543996
Chr5	30.787	Marker211088	89250399
Chr5	30.787	Marker211123	89356489
Chr5	31.25	Marker210376	79418405
Chr5	31.25	Marker210724	82768387
Chr5	31.715	Marker210606	80906734
Chr5	31.715	Marker210775	83129039
Chr5	32.412	Marker210797	83429435
Chr5	32.412	Marker210973	87544304
Chr5	33.032	Marker211145	89498962
Chr5	33.032	Marker211245	90021690
Chr5	33.53	Marker211148	89499150
Chr5	33.652	Marker211352	90483007
Chr5	34.272	Marker211297	90404387
Chr5	34.272	Marker211342	90482682
Chr5	34.272	Marker211478	91361452
Chr5	34.272	Marker211504	91687499
Chr5	34.272	Marker211878	95751565
Chr5	34.272	Marker211999	96800745
Chr5	35.147	Marker211941	96358489
Chr5	35.436	Marker212117	97586343
Chr5	35.436	Marker212286	98155192
Chr5	35.436	Marker212390	98587435
Chr5	35.436	Marker212473	99806844
Chr5	35.436	Marker212497	100071495
Chr5	35.745	Marker212518	100275251
Chr5	36.364	Marker212539	100536873
Chr5	36.364	Marker212560	100718911
Chr5	36.364	Marker212603	101348033
Chr5	36.364	Marker212742	102838618
Chr5	36.364	Marker212744	102865977
Chr5	36.364	Marker212810	104286532
Chr5	36.364	Marker212849	105002318
Chr5	36.364	Marker212970	106535601
Chr5	36.364	Marker212980	106536665
Chr5	36.364	Marker213060	106965501

Chr5	36.825	Marker213091	107208887
Chr5	37.299	Marker213219	108959524
Chr5	37.299	Marker213253	109967648
Chr5	37.764	Marker213306	110724951
Chr5	37.764	Marker213337	110829238
Chr5	38.229	Marker213404	111065185
Chr5	38.229	Marker213506	112366634
Chr5	38.229	Marker213550	113017659
Chr5	38.229	Marker213562	113044082
Chr5	38.229	Marker213694	113876079
Chr5	38.229	Marker213719	114031097
Chr5	38.229	Marker214079	117872092
Chr5	38.229	Marker214102	117972398
Chr5	38.694	Marker213190	108217634
Chr5	38.694	Marker213270	110196139
Chr5	38.694	Marker213273	110229871
Chr5	38.694	Marker213310	110746843
Chr5	39.681	Marker213812	115150140
Chr5	40.667	Marker214195	120067275
Chr5	40.667	Marker214251	120238562
Chr5	40.667	Marker214281	120420589
Chr5	42.648	Marker214301	120474650
Chr5	42.648	Marker214309	120529029
Chr5	43.634	Marker214366	121365103
Chr5	44.621	Marker214581	123906246
Chr5	44.621	Marker214635	124912616
Chr5	44.621	Marker214644	124935318
Chr5	44.852	Marker214838	125970170
Chr5	44.852	Marker214892	126493139
Chr5	44.852	Marker214912	126591633
Chr5	44.852	Marker215008	127662937
Chr5	44.852	Marker215018	127663219
Chr5	44.852	Marker215047	127702507
Chr5	44.852	Marker215072	127814104
Chr5	45.083	Marker214526	123588139
Chr5	45.083	Marker214818	125883696
Chr5	45.315	Marker214312	120573478
Chr5	45.315	Marker214361	121364824
Chr5	45.315	Marker214452	122622522
Chr5	45.315	Marker214471	122977950
Chr5	45.315	Marker214475	122978235
Chr5	45.315	Marker214476	122990446
Chr5	45.546	Marker214648	124944989

Chr5	45.546	Marker214825	125934829
Chr5	45.778	Marker214588	123914451
Chr5	45.778	Marker214663	125094954
Chr5	45.778	Marker214672	125175761
Chr5	45.778	Marker214784	125735822
Chr5	46.243	Marker214830	125935081
Chr5	46.708	Marker214695	125341884
Chr5	46.708	Marker214803	125827061
Chr5	47.409	Marker215037	127683432
Chr5	48.11	Marker215085	127999801
Chr5	48.11	Marker215132	128383744
Chr5	48.11	Marker215276	130234287
Chr5	48.11	Marker215281	130272566
Chr5	48.11	Marker215307	130466497
Chr5	48.514	Marker215476	131226972
Chr5	49.737	Marker215500	131436634
Chr5	49.737	Marker215599	132264928
Chr5	49.737	Marker216217	138603089
Chr5	50.311	Marker215418	130872893
Chr5	53.076	Marker216047	137139049
Chr5	54.498	Marker216006	136624823
Chr5	54.498	Marker216214	138602853
Chr5	55.437	Marker215491	131361262
Chr5	58.599	Marker215777	133827505
Chr5	58.599	Marker216225	138633464
Chr5	58.599	Marker216256	138878175
Chr5	58.599	Marker216265	138978538
Chr5	58.599	Marker216578	140606578
Chr5	60.979	Marker216576	140597784
Chr5	60.979	Marker216944	141823804
Chr5	62.151	Marker216400	139862480
Chr5	62.735	Marker216692	141336407
Chr5	62.934	Marker217322	144430196
Chr5	63.318	Marker217341	144458687
Chr5	63.318	Marker217490	146013669
Chr5	63.781	Marker217263	143810774
Chr5	63.781	Marker217457	145558383
Chr5	63.781	Marker217504	146161293
Chr5	64.07	Marker217646	147184816
Chr5	64.945	Marker217488	145977148
Chr5	64.945	Marker217693	147433372
Chr5	64.945	Marker218058	150213831
Chr5	64.945	Marker218398	152936648

Chr5	64.945	Marker218427	153112753
Chr5	65.176	Marker217192	143603284
Chr5	65.176	Marker218048	150170592
Chr5	65.454	Marker217896	148975622
Chr5	65.454	Marker217994	149891112
Chr5	66.578	Marker218577	154629639
Chr5	66.578	Marker218583	154630111
Chr5	66.578	Marker218633	155368546
Chr5	66.886	Marker218938	158216749
Chr5	67.516	Marker218691	155544210
Chr5	67.516	Marker218918	158107622
Chr5	69.429	Marker220030	166623185
Chr5	69.429	Marker220050	166713564
Chr5	69.429	Marker220066	166767240
Chr5	71.317	Marker219085	158987926
Chr5	71.317	Marker219243	160643124
Chr5	72.488	Marker219437	162180027
Chr5	72.488	Marker219479	162355926
Chr5	72.993	Marker218997	158447179
Chr5	72.993	Marker219064	158913843
Chr5	72.993	Marker219089	158988009
Chr5	72.993	Marker219371	161798081
Chr5	72.993	Marker219629	163683419
Chr5	73.347	Marker219527	162624548
Chr5	73.347	Marker219744	164491500
Chr5	73.775	Marker219808	165158515
Chr5	73.775	Marker220159	167135318
Chr5	73.775	Marker220251	168075643
Chr5	73.775	Marker220273	168222126
Chr5	73.775	Marker220296	168303953
Chr5	73.775	Marker220329	168663013
Chr5	74.203	Marker220359	168752863
Chr5	74.203	Marker220360	168753105
Chr5	75.062	Marker220750	175235275
Chr5	75.062	Marker221107	177768395
Chr5	75.49	Marker221153	177927380
Chr5	75.49	Marker221210	178312926
Chr5	77.225	Marker221745	182412304
Chr5	80.763	Marker222060	184965058
Chr5	80.763	Marker222393	187590872
Chr5	80.763	Marker222566	189219560
Chr5	83.483	Marker221054	177404241
Chr5	83.483	Marker221171	178171078

Chr5	83.844	Marker223043	192940599
Chr5	83.844	Marker223235	194488509
Chr5	83.844	Marker223295	194720470
Chr5	83.987	Marker221276	178436625
Chr5	83.987	Marker221373	179500894
Chr5	84.272	Marker223058	193285293
Chr5	84.272	Marker223139	193738136
Chr5	84.272	Marker223186	194361851
Chr5	86.451	Marker222690	190223080
Chr5	87.746	Marker222688	190222996
Chr5	90.374	Marker221914	184037746
Chr5	90.374	Marker221951	184466850
Chr5	90.374	Marker221958	184467129
Chr5	90.374	Marker222185	186156442
Chr5	91.304	Marker221569	181109991
Chr5	91.304	Marker221708	182241079
Chr5	91.304	Marker221883	183682654
Chr5	91.304	Marker222674	190150181
Chr5	91.767	Marker222668	190117373
Chr5	91.767	Marker222845	191447566
Chr5	92.697	Marker222703	190428399
Chr5	92.697	Marker223164	194144216

Supplementary table. S5 QTL mapping results for F1 progeny groups FN2021 and FN2023

Traits	QTL name	Chr	Left Marker	Right Marker	LLP	RLP	GD	LOD	PVE%
FN2021	*qFN5*_2021	5	Marker218938	Marker221210	66.886	75.490	8.60	5.83	11.80
FN2023	*qFN5*_2023	5	Marker217263	Marker221745	63.781	77.225	13.44	6.38	13.30

注：FN2021: fruit number, The number of fruits measured for the whole plant in 2021；FN2023：The number of fruits measured for the whole plant in 2023；QTL name: the name of quantitative trait locus ；Chr: chromosome；Left Marker: Significantly Associated Region Left End Starting Marker；Right Marker: Significant association area right end termination marker；LLP: left marker linkage map position, Starting marker genetic position from the left end；RLP: right marker linkage map position；GD: genetic distance；Unit: cM, centimorgan ；LOD: limit of detection；PVE%: phenotypic variation explained

Supplementary table. S6 Sequencing Data Statistics

**Sample**	**EW-2d**	**EW-7d**	**CM-2d**	**CM-7d**	**D-2d**	**D-7d**	**S -2d**	**S -7d**
Raw Reads	48961771	55216071	47831128	49156725	127904976	96371570	116595066	110273347
Clean Reads	46.8	53.2	46.4	47.6	123.3	92.5	112.4	104.8
Q20	97.78	97.77	97.81	97.76	97.49	97.49	97.53	97.68
Q30	93.93	93.89	93.94	93.88	93.28	93.32	93.4	93.73
GC Content%	44.69	44.95	44.60	44.86	44.92	45.12	44.85	44.67

Supplementary table. S7 Chromosome interval location information(Fig.(a))

CHROM	start	end	length	nSNPs
chr04	70393061	70960649	567588	4
chr05	115827199	121844150	6016951	183
chr05	148065332	173623217	25557885	652
chr05	186622315	192717337	6095022	27

Supplementary table. S8 Chromosome interval location information(Fig.(b))

CHROM	start	end	length	nSNPs
chr01	63222839	73751084	10528245	207
chr01	173337625	244240121	70902496	3617
chr02	32182678	43979109	11796431	918
chr03	112421318	136133011	23711693	344
chr03	146178247	201864494	55686247	1531
chr04	2012	72666536	72664524	2996
chr04	78178352	98120777	19942425	592
chr04	138675266	180966838	42291572	1658
chr05	49013130	58794364	9781234	215
chr05	94612941	213433720	118820779	3194
chr08	57660435	65152094	7491659	61
chr09	106352523	167450930	61098407	1308
chr09	169467743	196577638	27109895	1131
chr10	68531	12239402	12170871	734
chr11	98100397	103190952	5090555	212
chr14	144895536	154018714	9123178	186
chr15	581347	30195434	29614087	1332
chr15	49761232	64445767	14684535	465
